# Multiscale Computational Modeling of Vascular Adaptation: A Systems Biology Approach Using Agent-Based Models

**DOI:** 10.3389/fbioe.2021.744560

**Published:** 2021-11-02

**Authors:** Anna Corti, Monika Colombo, Francesco Migliavacca, Jose Felix Rodriguez Matas, Stefano Casarin, Claudio Chiastra

**Affiliations:** ^1^ Laboratory of Biological Structure Mechanics (LaBS), Department of Chemistry, Materials and Chemical Engineering “Giulio Natta”, Politecnico di Milano, Milan, Italy; ^2^ Institute for Chemical and Bioengineering, Department of Chemistry and Applied Biosciences, ETH Zürich, Switzerland; ^3^ Department of Surgery, Houston Methodist Hospital, Houston, TX, United States; ^4^ Center for Computational Surgery, Houston Methodist Research Institute, Houston, TX, United States; ^5^ Houston Methodist Academic Institute, Houston, TX, United States; ^6^ PoliTo^BIO^Med Lab, Department of Mechanical and Aerospace Engineering, Politecnico di Torino, Turin, Italy

**Keywords:** cardiovascular system, vascular remodeling, computer models and simulations, multiscale models, agent-based models (ABMs), continuum-based models, equation-based modeling

## Abstract

The widespread incidence of cardiovascular diseases and associated mortality and morbidity, along with the advent of powerful computational resources, have fostered an extensive research in computational modeling of vascular pathophysiology field and promoted *in-silico* models as a support for biomedical research. Given the multiscale nature of biological systems, the integration of phenomena at different spatial and temporal scales has emerged to be essential in capturing mechanobiological mechanisms underlying vascular adaptation processes. In this regard, agent-based models have demonstrated to successfully embed the systems biology principles and capture the emergent behavior of cellular systems under different pathophysiological conditions. Furthermore, through their modular structure, agent-based models are suitable to be integrated with continuum-based models within a multiscale framework that can link the molecular pathways to the cell and tissue levels. This can allow improving existing therapies and/or developing new therapeutic strategies. The present review examines the multiscale computational frameworks of vascular adaptation with an emphasis on the integration of agent-based approaches with continuum models to describe vascular pathophysiology in a systems biology perspective. The state-of-the-art highlights the current gaps and limitations in the field, thus shedding light on new areas to be explored that may become the future research focus. The inclusion of molecular intracellular pathways (e.g., genomics or proteomics) within the multiscale agent-based modeling frameworks will certainly provide a great contribution to the promising personalized medicine. Efforts will be also needed to address the challenges encountered for the verification, uncertainty quantification, calibration and validation of these multiscale frameworks.

## Introduction

**FIGURE 1:**
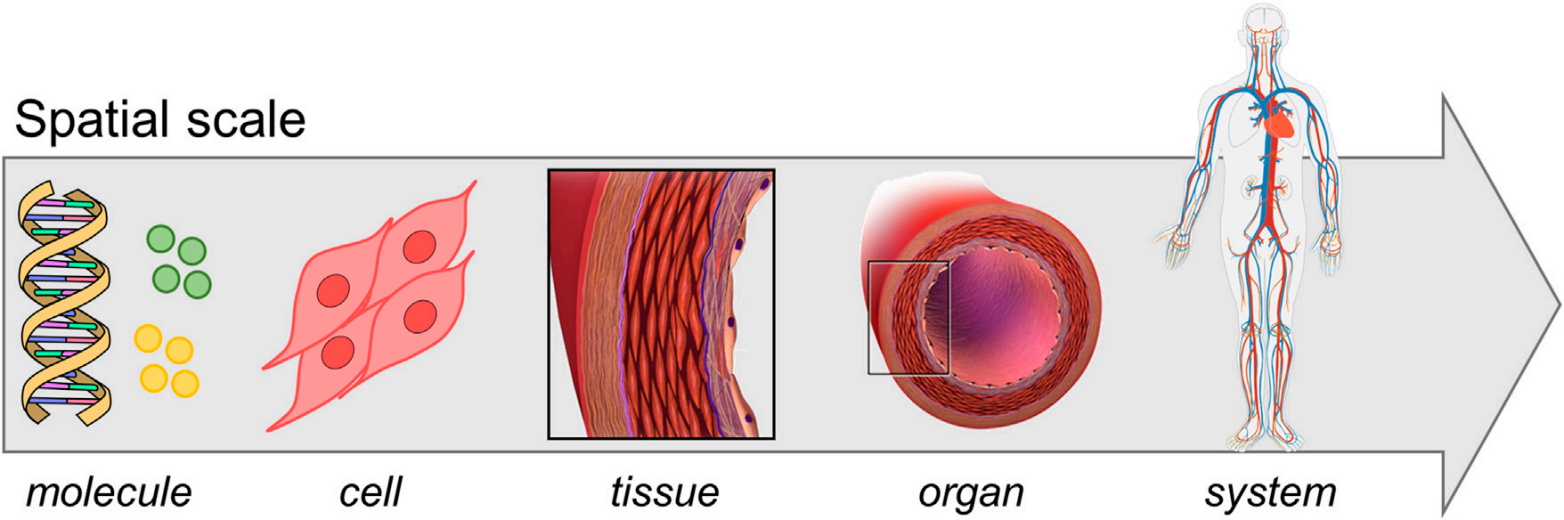
Multiscale vascular system. Adapted with permission from Wikimedia Commons, (public domain, https://commons.wikimedia.org/wiki/File:DNA_simple2.svg, https://commons.wikimedia.org/wiki/File:Circulatory_System_no_tags.svg), Blausen.com staff (2014) (https://creativecommons.org/licenses/by/3.0/), and from Blanco et al. (2017) (http://creativecommons.org/licenses/by-nc/4.0/).

In the past two decades the widespread incidence of cardiovascular diseases and associated mortality and morbidity (Virani et al., 2021), together with the increase in computer resources, promoted an extensive research in the field of vascular pathophysiology computational modeling. The vascular system lays on a hierarchical and multiscale structure (Qu et al., 2011) with different spatial and time scales involved in the pathophysiological processes (Figure 1): the molecular scale typically spans from nanoseconds to microseconds, the cellular one from seconds to hours, while the tissue/organ one from days to months (Walpole et al., 2013; Gosak et al., 2018). Processes at different scales influence each other through a complex network that includes heterogeneous mechanisms (e.g., mechanotransduction, gene pattern alteration) and ultimately leads to tissue and organ response (Kholodenko, 2006; Gosak et al., 2018). In vascular medicine, a thorough understanding of the complex network underlying vascular pathologies and the maladaptive healing processes in response to endovascular or surgical interventions is lacking. The analysis of the inter-scale interaction, from molecular pathways to pathological phenotype, is deemed crucial towards the delivery of personalized therapies and therefore it is receiving great interest (Kramer et al., 2018). Furthermore, since the shifting from reductionist to “systems biology” approach (beginning of the 21st century), a biological system is seen as a complex network involving environmental conditions, feedback mechanisms and mutual interactions across different scales, rather than as the mere sum of its components (Kohl et al., 2010; Mazzocchi, 2012; Kesić, 2016). Multiscale computational models are perfect tools for investigating these complex systems since they potentially embed the systems biology principles, making them suitable to bridge *in-vitro* models of single-scale phenomena to *in-vivo* models of a whole system of interest (Qu et al., 2011; Walpole et al., 2013). A systems biology approach allows tracking the propagation of a physical quantity across the multiscale network and quantifying its effect at tissue/organ level. This is fundamental to elucidate intracellular patterns, feedback mechanisms and cause-effect relations that are difficult to discern from *in-vitro* or *in-vivo* experiments, as well as from single-scale *in-silico* models (Qu et al., 2011). Such a level of detail offers a powerful instrument in the optic of personalized medicine, which is thought to revolutionize the therapeutic/diagnostic approach (Vogenberg et al., 2010). Accordingly, *in-silico* models are establishing to drive the biomedical research in a more robust fashion. This is supported by the progresses in biomedical technologies (e.g., imaging, high-throughput genomic sequencing) and the availability of high-performance computational resources, which allow elaborating huge quantity of data and integrating them in well-established computational infrastructures (Schadt et al., 2010; Hoekstra et al., 2019).

Two main modeling classes are adopted in the field of computational modeling of vascular pathophysiology, namely equation-based models and agent-based models (ABMs). Equation-based models are continuum models based on systems of ordinary differential equations (ODEs) or partial differential equations (PDEs). ODEs are used to describe the temporal variation of system state variables, while PDEs capture both temporal and spatial-related evolution of said variables. At the molecular level, PDE systems (e.g., advection-diffusion-reaction equations) are broadly implemented in the vascular field to describe the transport of molecular species (e.g., low density lipoproteins, inflammatory cytokines and other pro-atherogenic species (Silva et al., 2020)). In addition to transport phenomena, equation-based models are adopted to evaluate the mechanical behavior or the fluid dynamics at tissue/organ scale (e.g., to quantify stresses and strains in the arterial wall or the hemodynamic variables following endovascular procedures as percutaneous transluminal angioplasty (PTA) with/without stenting (Chiastra et al., 2021a; Chiastra et al., 2021b; Colombo et al., 2021a; Colombo et al., 2021b)). Usually, given the complex geometry of the vascular segments, numerical methods, such as finite difference method, finite element method (FEM) or finite volume method, are needed to solve PDE systems associated to solid mechanics or fluid dynamics problems (Walpole et al., 2013). ABMs are suitable tools to model heterogeneous populations and capture the behavior of systems with an intrinsic discrete nature, as systems of cells (Bonabeau, 2002; An et al., 2009). Moreover, ABMs effectively embed the systems biology approach: the system behavior emerges from the simulation of the 1) individual agent dynamics (e.g., cells), 2) interaction among agents and 3) environmental effects. Compared to continuum models, ABMs offer a natural description of cellular systems through the definition of rules governing the agent activities (e.g., mitosis, apoptosis (Hwang et al., 2009)). Thanks to this bottom-up approach, a complete understanding of the whole system is not needed, since its behavior will naturally emerge from the imposed basic rules. Moreover, while equation-based models tend to be mostly deterministic, ABMs can more easily incorporate stochasticity. Accordingly, multiple runs of the same ABM produce heterogeneous outputs, consistently with real observations of the phenomena, making ABMs closer to the reality. Finally, ABMs easily capture spatial-related aspects as tissue heterogeneity, composition and morphology, and can integrate phenomena at different scales within multiscale frameworks (Glen et al., 2019). Each modeling strategy introduced above allows simulating phenomena at specific tiers of resolution reaching a high-fidelity level. However, since biological processes involve different spatio-temporal scales, the integration of said tools into biological systems’ multiscale models is required (Walpole et al., 2013; Norton et al., 2019).

Recently, several multiscale models were proposed to capture the complex nature of vascular pathologies and depict the driving mechanisms of response to endovascular procedures or surgical interventions. The aim of the present review is to point out works on multiscale modeling of vascular remodeling, with special emphasis on those frameworks integrating continuum models and agent-based approaches in a systems biology perspective. Studies that proposed ABMs as the core of said frameworks are reviewed herein, highlighting the potentials of multiscale agent-based modeling methodology in incorporating the systems biology principles and capturing mechanobiological processes in vascular pathophysiology. In detail, the second section *(Agent-Based Modeling: Promising Tool for a Systems Biology Approach)*, provides a description of the ABM strategy, focusing on relevant aspects in the context of complex biological systems and multiscale approach. The third section *(Multiscale Agent-Based Modeling Frameworks of Vascular Pathophysiology)* describes the state-of-the-art of computational multiscale agent-based modeling framework of vascular pathophysiology. Specifically, models of atherosclerosis, in-stent restenosis (ISR) and vein graft adaptation will be detailed, as well as studies focusing on other aspects of vascular remodeling processes. The fourth section *(Agent-Versus Continuum-Based Multiscale Frameworks: Strengths and Limitations)* discusses the strengths and limitations of agent-versus continuum-based frameworks. The subsequent section *(Challenges and Future Directions)* presents the current challenges of agent-based modeling strategies and future perspectives in the field, while the last section *(Conclusion)* the concluding remarks.

## AGENT-BASED MODELING: PROMOSING TOOL FOR A SYSTEMS BIOLOGY APPROACH

ABMs belong to a class of computational models in which the system of interest is replicated with a bottom-up approach, i.e. through the discrete representation of its components, called “agents”, as autonomous decision-making elements (Bonabeau, 2002; An et al., 2009). The behavior of each agent is described through sets of rules, which can be either probabilistic or deterministic and may depend on internal and external variables: the former account for the intrinsic dynamics of the agent and the latter for the effects of the surrounding environment and neighboring agents (Bonabeau, 2002; An et al., 2009). Doing so, the system behavior is not reduced to the mere superimposition of its elementary components but it emerges from the concurrent agent actions, interactions, mutual influence with the environment, and feedback loops that dynamically evolve throughout the simulation (Chavali et al., 2008). Consequently, a simple ABM can give rise to complex, non-linear phenomena that are counterintuitive or difficult to predict from the analysis of its elementary components’ behavior (emergent properties) (An et al., 2009).

**FIGURE 2:**
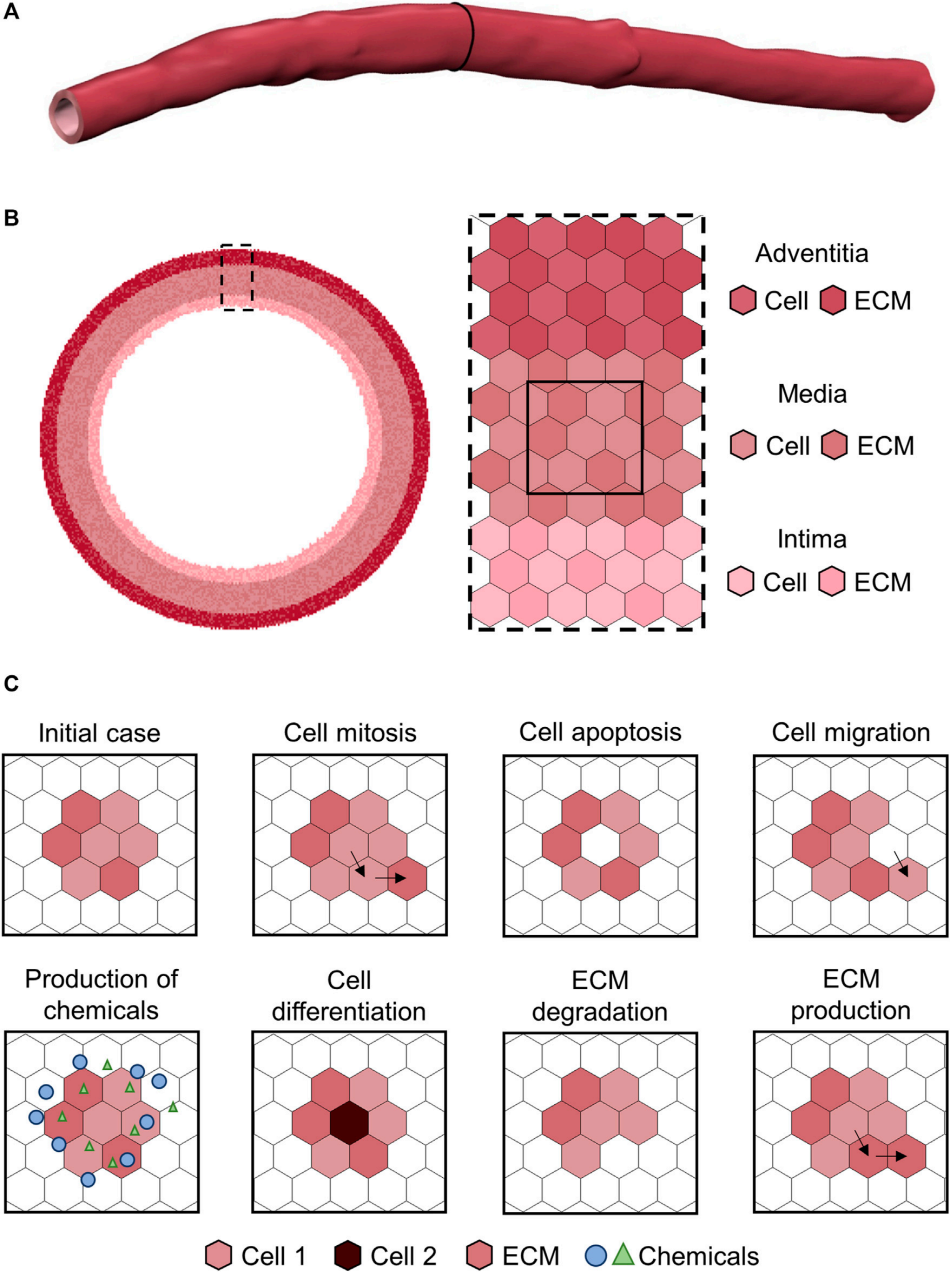
Example of agent-based model (ABM) of vascular wall at the cell-tissue scale implemented on a hexagonal lattice, as in Corti et al. (2020). **(A)** Three-dimensional patient-specific vessel geometry. **(B)** Cell-tissue scale ABM of a vessel cross-section implemented on a hexagonal lattice. The vessel wall is composed by the intima, media and adventitia layers. Each layer is populated by cell and extracellular matrix (ECM) agents, with each agent occupying one lattice site. **(C)** Examples of ABM rules.

Considering all the above, ABMs provide a simple but effective and realistic representation of systems composed by heterogeneous populations of active elements, in which the interactions and the spatial-related aspects play a major role. Translating these concepts to biology, ABMs present great potentialities in modeling complex biological processes through an intuitive and flexible framework (Bonabeau, 2002; An et al., 2009). Moreover, the basic principles of these models make them suitable to express the systems biology approach, since the concepts of emergence and the holistic representation of systems are naturally implemented (Kohl et al., 2010). The most common scale of representation of biological systems through ABMs is the cell-tissue level, in which each cell or extracellular matrix (ECM) component constitutes an autonomous agent (Figure 2) (Hwang et al., 2009). Cellular dynamics are replicated with dedicated rules (Hwang et al., 2009), along with other phenotype-specific events (e.g., production of chemicals, intracellular signaling (An et al., 2009)). As mentioned above, all these rules can be an explicit function of variables representing the concentration of chemicals (e.g., drugs), local microenvironment (e.g., hypoxia, inflammation), mechanical stimuli (e.g., state of stress), and agent-specific internal conditions (e.g., cell cycle period) (Van Liedekerke et al., 2015). In such a scenario, rule-based approaches are more intuitive than differential equations-based systems, especially for non-mathematicians (Bonabeau, 2002), and this is a crucial aspect in the era of multidisciplinary research. Accordingly, ABMs have the potential to abate the background-derived roadblocks preventing biologists/clinicians from translating their conceptual model of the biological process into an *in-silico* replica for further advancing their research.

Different strategies are adopted to implement agent rules. Among them, “if-then” conditions are often used to express different behaviors according to specific situations (Bonabeau, 2002). Cell proliferation usually occurs only if specific conditions hold, e.g., if the cell is in the mitotic phase, if there is physical space for the new cell according to the contact inhibition criterion, if inhibitory signals are deactivated (Hwang et al., 2009). In addition, force-based or energy-based criteria can be adopted to define agent and system equilibrium conditions (Zun et al., 2017). Moreover, deterministic or stochastic rules can be used. Stochastic rules are commonly implemented to incorporate an intrinsic level of randomness (e.g., in the form of noise, random switching between different states of the system (Andrews et al., 2009)) that allows generating a population of outputs consistent with the statistical observation of the real phenomenon (An et al., 2009). Repeated simulations of the same ABM (under identical initial and boundary conditions) will exhibit different behaviors, resulting in multiple possible evolutions of the system. This well reproduces the reality of biological processes observed at a population level: for example, the heterogeneous outcomes of *in-vitro* and *in-vivo* experiments, as well as of clinical trials and, more in general, the inter-subject variability encountered in any clinical study. The embedded stochasticity may also lead to unexpected and rare event combinations resulting in an unusual system evolution that, although constituting an outlier from a statistical viewpoint, may highlight counterintuitive and unpredictable processes that may realistically occur. The stochasticity does not necessarily represent a real stochastic event in the biological system (An et al., 2009). In some cases, even though the underlying processes may be intrinsically deterministic, it can be advantageous describing the event itself as stochastic and based on a specific probability density function derived from observations of the exhibited phenomenon (An et al., 2009; Székely and Burrage, 2014). For example, cell proliferation is driven by a cascade of deterministic sub-processes. However, if a detailed knowledge of all the sub-processes is lacking or if their explicit modeling is beyond the purpose of the work, the final event (i.e., cell proliferation) can be replicated through a probabilistic rule that follows a phenomenologically-derived probability density function.

**FIGURE 3:**
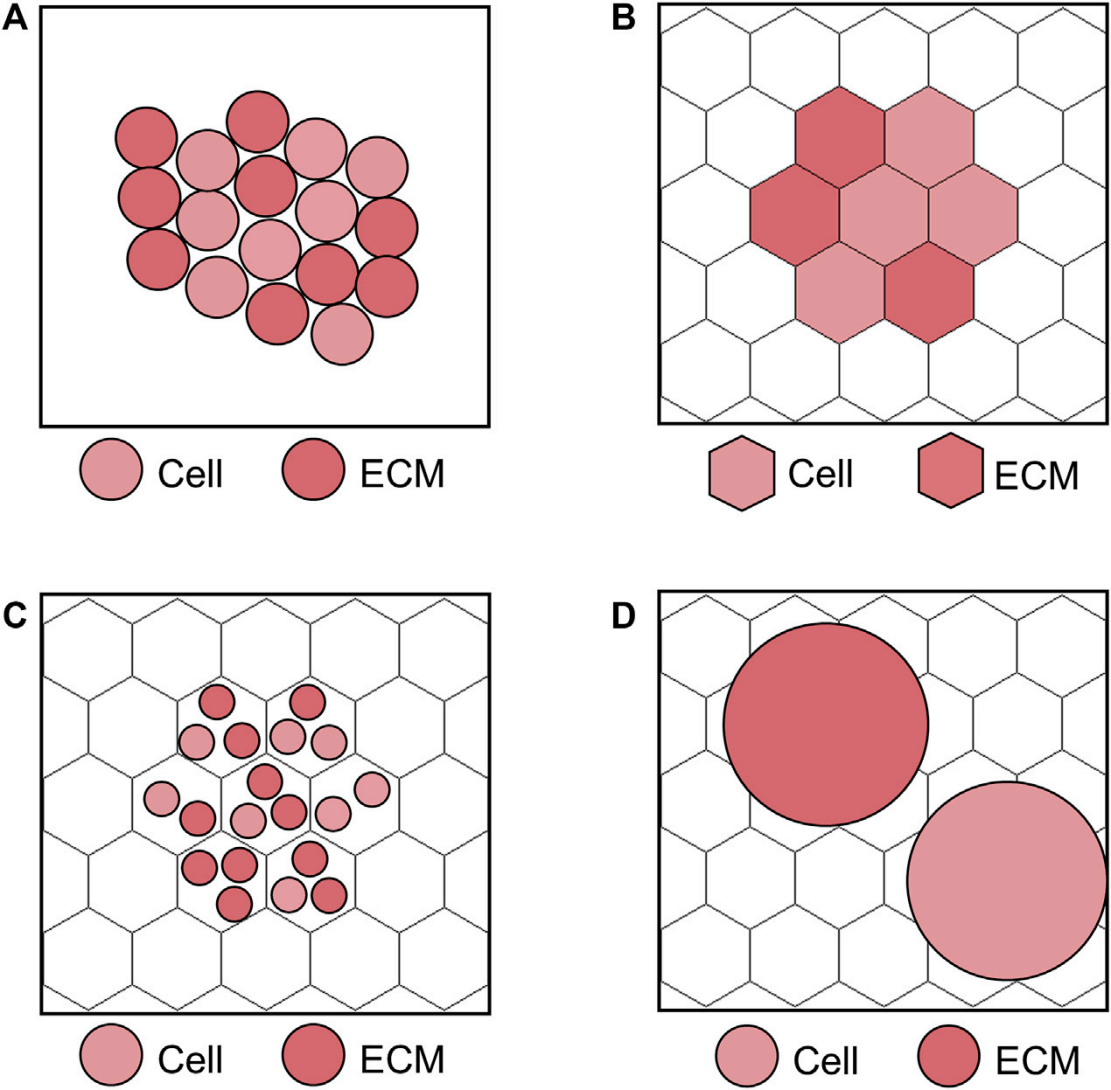
Examples of lattice-free and lattice-based agent-based model (ABM) at the cell scale. **(A)** Lattice-free ABM. **(B)** Lattice-based ABM with one lattice site corresponding to a single agent, i.e., cell or extracellular matrix (ECM). **(C)** Lattice-based ABM with multiple agents (cells and ECM) at one lattice site. **(D)** Lattice-based ABM with agents (cells and ECM) occupying more than one lattice site. The lattice-based ABMs shown in **(B–D)** are implemented on a hexagonal lattice, as in Corti et al. (2020).

ABMs mainly divide in two classes depending on their implementation on a lattice (lattice-based) or in the continuum (lattice-free) space, as schematized in Figure 3 (Van Liedekerke et al., 2015). Within lattice-based ABMs, the choice of the relative agent dimension with respect to the lattice site may vary according to the model purposes (Van Liedekerke et al., 2015). Focusing as example on the cell level scale, the following three different strategies are possible (Van Liedekerke et al., 2015): 1) one lattice site corresponds to a single cell (Figure 3B), 2) one lattice site contains multiple cells (Figure 3C) or 3) many lattice sites are occupied by a cell (Figure 3D). The strategies 1) and 2) are adopted when large systems of cells are simulated and attention is given to cell activities (resulting in the evolution of the system), rather than on local cell deformation processes. The choice of 1) or 2) has minor effects on results accuracy, but mainly affects the computational time. Differently, the strategy 3) is preferred if the local effects on cells (e.g., the explicit representation of cell shape and deformation) are of interest and a small cell system is considered, thus making it more suitable for processes as tumor growth or angiogenesis. Since in lattice-free ABMs agents can occupy any position in the continuum space, these are usually the first choice if the interactions among cells are pivotal to study the trajectory of the system of interest (Randles et al., 2021). They are typically embedded with immersed boundary techniques to describe the mutual agent interaction (Garbey et al., 2019) and usually force-based or energy-based equations are solved to compute agent movement (Van Liedekerke et al., 2015). However, a fine detail in this direction comes with the price of a higher computational cost.

ABMs are flexible and modular (Bonabeau, 2002; An et al., 2009). Once the structure of the model is implemented, the inclusion of new agent types (with their rule set) or new events for existing agents is still possible and does not affect the general body of the model. Accordingly, increasing levels of complexity can be explored through a stepwise process and a modular framework can be adopted, with the possibility to switch on/off processes according to the goal of the planned simulation. As downsides, 1) integrating an existent model with additional components might implicate a re-calibration of the model if some coefficients of the new agents are unknown or not directly retrievable from dedicated experimental data, and 2) the more the complexity of the model increases, the more the model becomes unmanageable and unusable in practice. Thanks to their flexibility, cell-scale ABMs can be coupled with continuum models at tissue or molecular scale, leading to multiscale agent-based modeling frameworks of biological systems, in which the ABM constitutes the main core (An et al., 2009; Van Liedekerke et al., 2015). A bidirectional interaction between the ABM and the continuum modules simulates the influence that the external environment (at the tissue or molecular scales) has on cellular dynamics and vice-versa. This allows capturing the adaptation of cell behavior in response to molecular or mechanical factors from one side, and the environment modification as consequence of cell activities from the other side. Many studies have demonstrated the potential of multiscale agent-based modeling frameworks to model biological systems in different areas of applications, such as tissue remodeling (e.g., Rouillard and Holmes (2014), Virgilio et al. (2015) and Chen et al. (2018)), tumor growth (e.g., Wang and Vafai (2015) and Norton et al. (2019)) and wound healing (e.g., Mi et al. (2007), Dutta-Moscato et al. (2014) and Rikard et al. (2019)). In addition, the application of similar multiscale frameworks for modeling vascular adaptation processes is emerging, as extensively discussed in the following section.

## MULTISCALE AGENT-BASED MODELING FRAMEWORKS OF VASCULAR PATHOPHYSIOLOGY

Multiscale agent-based modeling frameworks of vascular pathophysiology have been developed by several research groups to predict vessel response to the alteration of the environmental and operational conditions and to provide insights into the driving mechanisms of post-intervention vascular remodeling at different temporal and spatial scales. To date, the most relevant applications of these computational frameworks regarded the atherosclerotic plaque development and the processes of restenosis following endovascular procedures and of vein graft neointimal hyperplasia after bypass surgery, as described in detail in, *Multiscale Models of Atherosclerosis* and *Multiscale Models of In-Stent Restenosis*, respectively. Furthermore, other vascular remodeling processes have been modeled (i.e., the arterial response to the alteration of growth factors, chemicals or mechanical stimuli and the remodeling process of a vascular tissue engineering scaffold), as reviewed in *Multiscale Models of Other Vascular Applications*.

**FIGURE 4:**
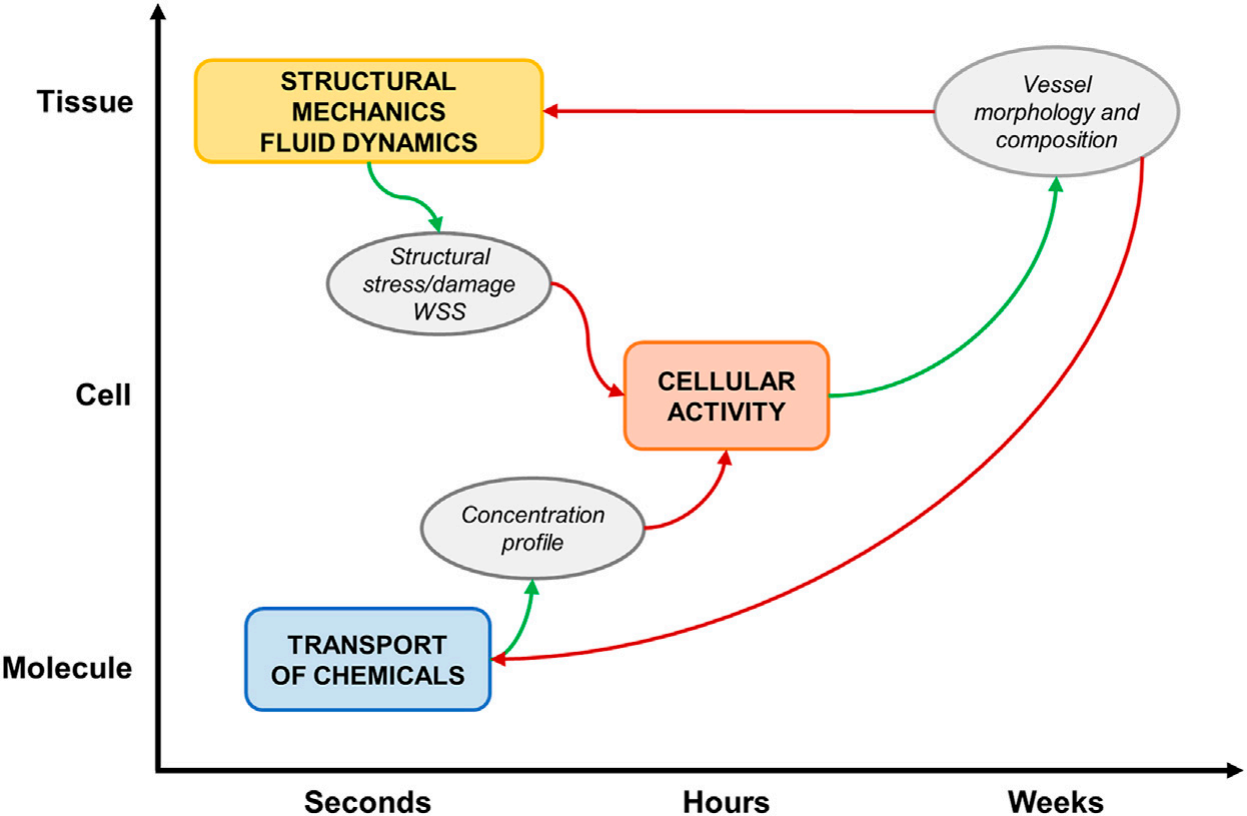
Schematic representation of a general multiscale framework of vascular adaptation. The main simulated events at the different spatio-temporal scales are: 1) structural mechanics and fluid dynamics, 2) cellular activity and 3) molecular transport. The modules receive proper inputs (red arrows) and generate suitable outputs (green arrows).

A general computational approach characterizes most of the reviewed studies (Figure 4), based on a framework integrating: 1) a tissue-scale module (e.g., vascular segment), which simulates the hemodynamics and/or the solid mechanics, usually with a continuum approach; 2) a cell-scale module (e.g., vascular cells and ECM), which replicates cellular activities in response to hemodynamic, mechanical, chemical stimuli with a discrete approach (i.e., ABM) and 3) a molecular-scale (subcellular) module, which computes the transport of molecules (e.g., growth factors, chemicals, drugs) within the tissue or simulates the expression profile of proteins and genes with a continuum approach.

The ABM is the core of the multiscale framework. It is initialized with hemodynamic, mechanical or molecular cues and simulates vascular remodeling by implementing cellular behaviors. The morphological and compositional tissue changes, resulting from the ABM simulation, are used to update the tissue and molecular scale configurations, which undergo new simulations to compute the updated conditions for the ABM re-initialization.

### Multiscale Models of Atherosclerosis

Atherosclerosis is a multifactorial and inflammatory-driven disease that leads to the narrowing of the arterial lumen due to the formation of a plaque in the arterial wall (Bentzon et al., 2014). The early pathological onset was attributed to the accumulation of circulating low-density lipoproteins (LDL) in the arterial wall, which, by triggering an inflammatory response and a subsequent network of cause-effects events (involving e.g., monocytes recruitment, LDL oxidation, foam cell accumulation, fatty streaks formation, smooth muscle cell (SMC) increased synthetic activity), ultimately promotes atherosclerotic plaque formation (Libby et al., 1992; Bentzon et al., 2014). The lipid-rich plaque may also progress into advanced atherosclerotic lesion, characterized by necrotic core formation, fibrosis and calcification (Bentzon et al., 2014). The initial trigger of the pathology, namely the increased endothelial permeability to LDL, facilitating LDL accumulation in the intima, is associated with endothelial dysfunction, which is promoted by several factors as diabetes, hypercholesterolemia, hypertension, smoking and obesity (Mudau et al., 2012). Moreover, evidence of co-localization of plaque formation and luminal regions exposed to altered hemodynamics, characterized by low and/or oscillatory wall shear stress (WSS), suggested an implication of disturbed blood flow in the development of the pathology (Chatzizisis et al., 2007; Samady et al., 2011). Specifically, the exposure of endothelial cells to disturbed blood flow triggers an intracellular signaling pathway that reduces endothelial nitric oxide synthase expression and the nitric oxide bioavailability, promoting increased SMC synthetic activity and the activation of atherogenic processes (Harrison et al., 2006).

Some of the aforementioned aspects of pathology initiation and progression were considered in the available multiscale agent-based modeling frameworks of atherosclerosis, as presented in Table 1 and Supplementary Tables S1, S2, and discussed below. Table 1 describes the pathology, the framework, the agent types and the computational domain considered in each study. Supplementary Table S1 details the module integration and the software, while Supplementary Table S2 the ABM strategies, namely the vessel wall compartments, and the agent types and rules.

**TABLE 1:** Multiscale agent-based modelling frameworks of vascular adaptation.

Authors (Year)	Pathology	Multiscale framework	Agents	Domain
Bhui and Hayenga (2017)	Atherosclerosis	**Tissue-scale module** (seconds)	EC, SMC (inert agents), Leukocytes (Neutrophils, monocytes, macrophages, foam cells and lymphocytes)	Simplified 3D model of coronary artery
		Hemodynamics module: FEM. I: vessel geometry; O: WSS		
		**Cell-scale module** (hours/days)		
		ABM; I: WSS; O: vessel geometry and wall composition		
		**Molecular-scale module** (seconds)		
		Cytokine and LDL transport in the ABM		
Corti et al. (2019), Corti et al. (2020)	Atherosclerosis	**Tissue-scale module** (seconds)	SMC, ECM (collagen, elastin), LDL, Fibroblasts	Idealized 3D model of superficial femoral artery, with 2D ABM cross-sections
		Hemodynamics module: FVM. I: vessel geometry; O: WSS		
		**Cell-scale module** (hours/days)		
		ABM; I: WSS; O: vessel geometry and wall composition		
Caiazzo et al. (2011); Tahir et al. (2011); Tahir et al. (2013); Tahir et al. (2014); Zun et al. (2017); Zun et al. (2019)	In-stent restenosis	**Tissue-scale module** (seconds)	SMC, IEL (Caiazzo et al. (2011); Tahir et al. (2011); Tahir et al. (2013); Tahir et al. (2014)); SMC, IEL, EEL (Zun et al. (2017)); SMC, ECM, IEL, EEL (Zun et al. (2019))	2D longitudinal section of idealized straight artery with 2 stent struts (Caiazzo et al. (2011)),Tahir et al. (2011); Tahir et al. (2013) - 6 stent struts (Tahir et al. (2014)); 3D straight artery (Zun et al. (2017)); Idealized curved artery with stent reconstructed from micro-CT. (Zun et al. (2019))
		Hemodynamics module: Lattice Boltzmann. I: vessel geometry; O: WSS/OSI		
		**Cell-scale module** (hours/days)		
		ABM - physical solver: stent deployment and structural cell dynamics. I: vessel geometry; O: equilibrium position, vessel geometry and structural stress		
		ABM - biological solver: SMC cell-cycle. I: WSS/OSI, drug concentration, structural stress. O: vessel geometry		
		**Molecular-scale module** (seconds)		
		Drug diffusion: FD. I: vessel geometry; O: drug concentration in the tissue. Included in (Caiazzo et al. (2011); Tahir et al. (2011))		
Boyle et al. (2010)	In-stent restenosis	**Tissue-scale module** (seconds)	SMC, EC ECM, matrix degrading factors and growth factors modeled as agent internal variables	Solid mechanics module: artery as 3D cylinder (symmetry: 1/8^th^ model circumferentially). Lattice-based model: 2D longitudinal section
		Solid mechanics module: FEM. Stent expansion. I: vessel geometry; O: vessel geometry, minimum principal stress		
		**Cell-scale module** (hours/days)		
		ABM. I: vessel geometry, minimum principal stress. O: updated vessel geometry and wall composition		
Boyle et al. (2011)	In-stent restenosis	**Tissue-scale module** (seconds)	SMC ECM, matrix degrading factors, growth factors and damage modeled as agent internal variables	2D cross-section of an ideal cylindrical artery with 6 stent struts. 1/6^th^ of the model considered for symmetry
		Solid mechanics module: FEM. Stent expansion. I: vessel geometry; O: vessel geometry, von Mises stress		
		**Cell-scale module** (hours/days)		
		ABM. I: vessel geometry, damage, matrix degrading factors, growth factors, ECM; O: updated vessel geometry and wall composition		
		**Molecular-scale module** (seconds)		
		Inflammation module: Set of ODEs. I: von Mises stress. O: damage, matrix degrading factors, growth factors, ECM		
Zahedmanesh et al. (2014)	In-stent restenosis	**Tissue-scale module** (seconds)	SMC, EC ECM, matrix degrading factors and damage modeled as agent internal variables	2D longitudinal section (axisymmetric model) of artery and single stent strut
		Solid mechanics module: FEM. Stent expansion. I: vessel geometry; O: vessel geometry, von Mises stress		
		**Cell-scale module** (hours/days)		
		ABM. I: vessel geometry, damage (sigmoid function of von Mises stress); O: updated vessel geometry and wall composition		
Nolan and Lally (2018)	In-stent restenosis	**Tissue-scale module** (seconds)	SMC, EC, ECM, matrix degrading factors, growth factors, phenotype and damage modeled as agent internal variables	2D quarter cylinder of artery in the radial-circumferential plane
		Solid mechanics module: FEM. Stent expansion. I: vessel geometry; O: vessel geometry, von Mises stress		
		**Cell-scale module** (hours/days)		
		ABM. I: vessel geometry, damage, matrix degrading factors, growth factors, ECM, phenotype; O: updated vessel geometry and wall composition		
		**Molecular-scale module** (seconds)		
		Inflammation module: Set of ODEs. I: von Mises stress. O: damage, matrix degrading factors, growth factors, ECM, phenotype		
Li et al. (2019)	In-stent restenosis	**Tissue-scale module** (seconds)	SMC, EC ECM, matrix degrading factors, growth factors, cell phenotype and damage modeled as agent internal variables	2D longitudinal section (axisymmetric model) of artery and single stent strut
		Solid mechanics module: FEM. Stent expansion and structural equilibrium following geometrical changes. I: vessel geometry; O: vessel geometry, von Mises stress		
		**Cell-scale module** (hours/days)		
		ABM. I: vessel geometry, damage, matrix degrading factors, growth factors, ECM; O: updated vessel geometry and wall composition		
		**Molecular-scale module** (seconds)		
		Inflammation module: set of ODEs. I: von Mises stress. O: damage, matrix degrading factors, growth factors, ECM, cell phenotype		
Garbey et al. (2015)	Vein graft remodeling	**Tissue-scale module** (seconds)	SMC, ECM	2D circular vein graft model
		Hemodynamics module: FVM and immersed boundary implementation. I: vessel geometry; O: WSS.		
		Solid mechanics module: FEM. I: vessel geometry; O: loaded vessel geometry, wall tension		
		**Cell-scale module** (hours/days)		
		ABM. I: WSS, wall tension; O: updated unloaded vessel geometry and wall composition		
Garbey et al. (2017)	Vein graft remodeling	**Tissue-scale module** (seconds)	SMC, ECM	2D circular vein graft model
		Hemodynamics module: Analytical solution (Poisson problem). I: vessel geometry; O: WSS.		
		Solid mechanics module: Analytical solution thick wall cylinder. I: vessel geometry; O: wall tension		
		**Cell-scale module** (hours/days)		
		ABM. I: WSS, wall tension; O: updated vessel geometry and wall composition		
Garbey et al. (2019)	Vein graft remodeling	**Tissue-scale module** (seconds)	SMC, ECM	2D circular vein graft model
		Hemodynamics module: Analytical solution (Poisson problem). I: vessel geometry; O: WSS.		
		Solid mechanics module: Analytical solution thick wall cylinder. I: vessel geometry; O: wall tension		
		**Cell-scale module** (hours/days)		
		ABM. SMC/ECM activities. I: WSS, wall tension; O: updated vessel geometry and wall composition		
		IBM. SMC migration and wall remodeling. I: ABM vessel geometry; O: updated vessel geometry and composition		
		**Molecular-scale module** (seconds)		
		Diffusion of growth factor. PDE. I: WSS; O: spatio-temporal evolution of growth factor		
Zahedmanesh and Lally (2012)	Remodeling of a vascular tissue-engineered scaffold	**Tissue-scale module** (seconds)	SMC, ECM	2D longitudinal section (axisymmetric model) of vascular scaffold
		Solid mechanics module: FEM. I: vessel geometry and wall composition; O: vessel geometry, cyclic strain, pore fluid velocity		
		**Cell-scale module** (hours/days)		
		ABM. I: vessel geometry, cyclic strain, pore fluid velocity. O: updated vessel geometry and wall composition		
Keshavarzian et al. (2018)	Arterial growth and remodeling under different conditions: growth factors, chemicals, blood pressure	**Tissue-scale module** (seconds)	EC, SMC, fibroblasts, ECM	3D model of coronary artery
		Solid mechanics module: FEM. I: vessel geometry and wall composition (use of a content-based strain energy density function); O: maximum principal stress and strain under different loading condition		
		**Cell-scale module** (hours/days)		
		ABM. I: vessel geometry, stress, strain. O: updated vessel geometry and wall composition		

ABM: agent-based model; FEM: finite element method; FVM: finite volume method; FD: finite difference; ODE: ordinary differential equation; PDE: partial differential equation; IBM: immersed boundary method; I: input; O: output; WSS: wall shear stress; OSI: oscillatory shear index; SMC: smooth muscle cell; EC: endothelial cell; ECM: extracellular matrix; LDL: low density lipoprotein; IEL: internal elastic lamina; EEL: external elastic lamina; 2D: bidimensional; 3D three-dimensional.

The available multiscale models of atherosclerosis (Bhui and Hayenga, 2017; Corti et al., 2019; Corti et al., 2020) captured the mutual influence between hemodynamics and arterial wall remodeling during atherogenesis and plaque development. The multiscale frameworks of Bhui and Hayenga (2017), Corti et al. (2019) and Corti et al. (2020) were based on the bidirectional coupling of a stochastic ABM of cellular dynamics and a hemodynamics module for blood flow computation. Additionally, in the framework of Bhui and Hayenga (2017) a molecular module was included to describe the transport of inflammatory cytokines and LDL within the arterial wall.

**FIGURE 5:**
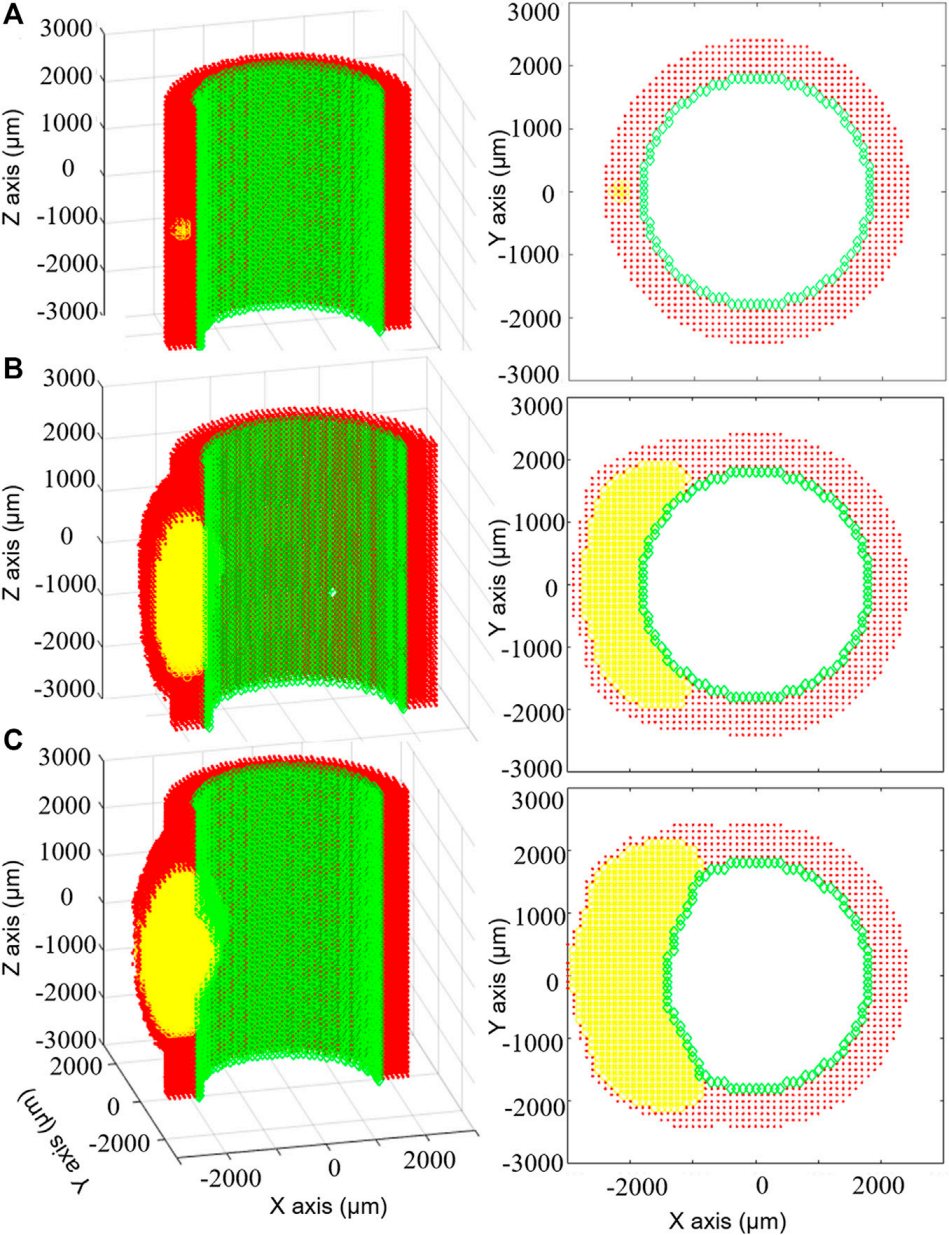
Results of the multiscale computational fluid dynamics–agent-based model (CFD-ABM) framework of atherosclerosis by Bhui and Hayenga (2017). The temporal evolution of the ABM geometry from the initial configuration **(A)** to the configuration at 6 months **(B)** and at 7 months **(C)** is shown, for the longitudinal **(left)** and transverse **(right)** views, with the endothelial cells in green, the arterial cells in red and leukocytes in yellow. The initial condition **(A)** is characterized by the presence of 15 leukocytes in the arterial wall. Until 6 months **(B)**, thanks to the compensatory Glagov’s remodeling, the plaque growth determined an outward remodeling, while preserving the lumen area. At this point, the plaque area is 40%. At 7 months **(C)**, the plaque growth provokes a reduction of the lumen area. Reprinted with permission from Bhui and Hayenga (2017) (http://creativecommons.org/licenses/by/4.0/).

The work by Bhui and Hayenga (2017) was applied to a three-dimensional (3D) idealized coronary artery model and investigated the role of WSS in the processes of leukocyte *trans*-endothelial migration, LDL accumulation and, consequently, atherosclerotic plaque progression. Computational fluid dynamics (CFD) simulations were performed to compute the WSS profile, used to initialize the ABM. Given the ABM-simulated changes of luminal geometry occurring during plaque growth, an ABM to CFD coupling was performed to update the WSS distribution, by computing the hemodynamics in the current vessel geometry. The ABM to CFD coupling occurred after significant changes in the luminal geometry rather than at a fixed time. A 3D ABM, constituted by a uniform layer for the arterial wall (i.e., without intima, media or adventitia separation) covered by a single layer of endothelial cells, was implemented. Leukocytes were the only active agents and specific rules for the endothelial adhesion, the *trans*-endothelial migration, the chemotactic migration in the arterial wall, the cytokines production, and the lifespan were implemented. In particular, leukocyte adhesion probability was defined as a function of WSS, circulating cytokine and leukocyte concentration, while the *trans*-endothelial migration as a function of arterial stiffness. Moreover, LDL transport and accumulation in the arterial wall depended on WSS and systemic LDL concentration. LDL diffusion in the arterial wall was modeled through Fick’s law and rules defining LDL oxidation and phagocytosis by monocyte-derived foam cells were applied. Finally, Glagov’s remodeling was implemented, according to which the lumen area was preserved in the initial phases of atherosclerosis thanks to a compensatory outward remodeling (Glagov et al., 1987). An example of the simulated plaque progression is provided in Figure 5. According to Glagov’s hypothesis, an outward enlargement of the arterial tissue was produced until month 6 of simulation, without affecting the lumen area. Then, further plaque growth led to lumen area decrease. Consistently with experimental and clinical findings, the framework simulated plaque growth at luminal regions exposed to low WSS (Stone et al., 2012). Additionally, the simulated mean lesion area was compared with observations in pig models of atherosclerosis (Pelosi et al., 2014). While a good agreement was found at 2 months, an underestimation of the plaque area was observed at 4 months, compared to animal data. This was attributed by the authors to the lack of SMC and fibroblast dynamics (migration, proliferation and ECM synthesis) in the model, which, if included, would have contributed to the lumen area reduction. Although the promising computational results, the assessment of their validity for human cases is challenging, due to the paucity of human data of early atherosclerosis. If translated to human cases, the simulated growth rate, may result accelerated. This was attributed by the authors to the use of *in-vitro* data for the conceptualization of leukocyte adhesion and migration rules and to the lack of ECM degradation processes. Considering all the above, it would be of great interest to include SMC and ECM dynamics to better appreciate their influence on plaque development.

**FIGURE 6:**
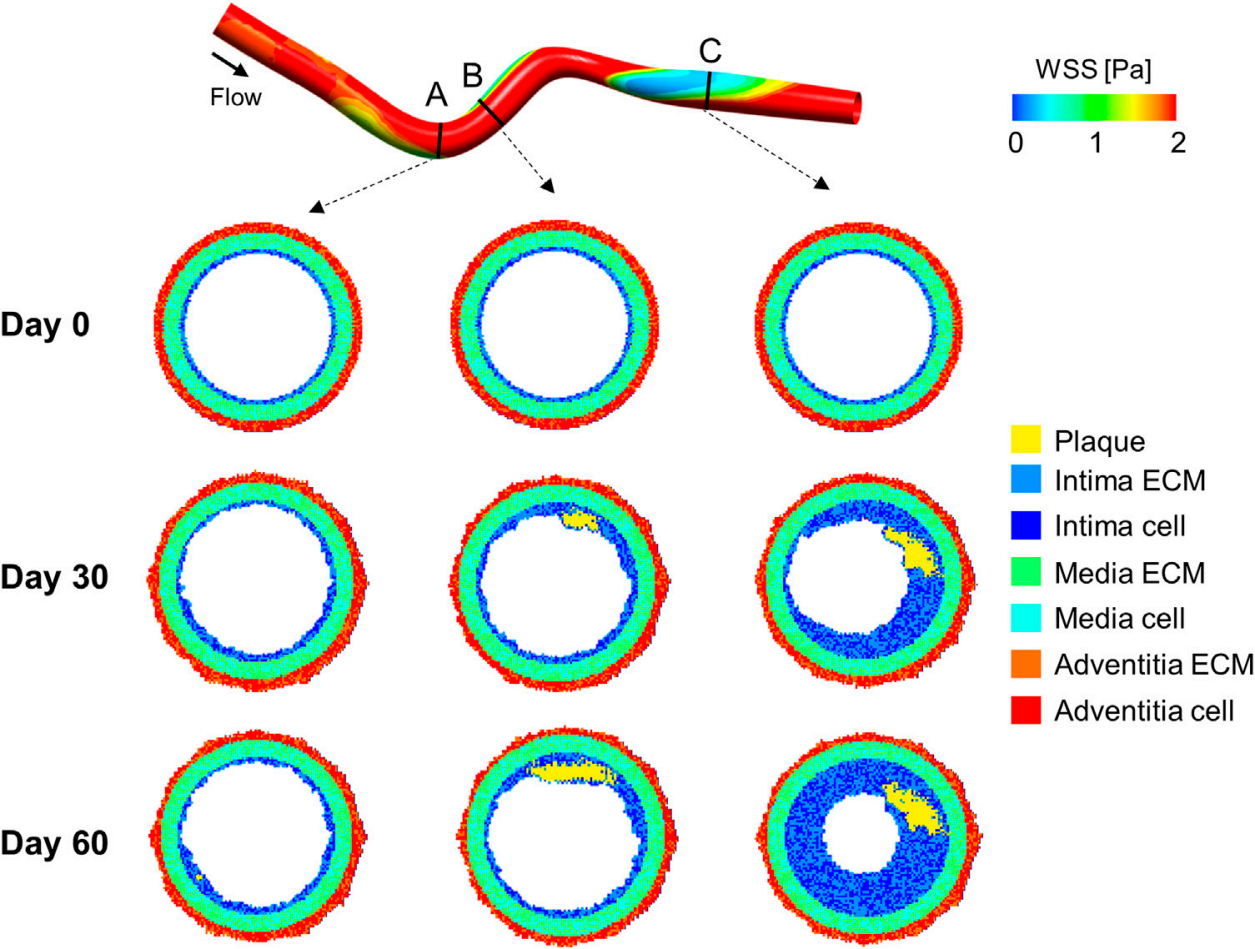
Results of the computational fluid dynamics–agent-based model (CFD-ABM) framework of atherosclerosis by Corti et al. (2020). At the top, the idealized 3D geometry of a superficial femoral artery is represented, with the wall shear stress (WSS) luminal distribution computed from steady-state CFD simulation. At the bottom, the ABM temporal evolution of three representative cross-sections is presented at day 0, 30 and 60. Greater plaque formation and lumen area reduction is obtained at luminal regions exposed to low WSS, such as downstream from the curvature (planes B and C). The planes involved in the atherogenic process (planes B and C) present an asymmetric plaque characterized by the presence of a well-defined lipid core (in yellow) and an increased intimal extracellular matrix (ECM) and smooth muscle cell (SMC) content (blue and light-blue, respectively).

A multiscale CFD-ABM framework was also proposed by Corti et al. (2019) and Corti et al. (2020). Steady-state CFD simulations of a 3D idealized superficial femoral artery model were coupled with a 2D ABM of cellular dynamics implemented for 10 evenly spaced vessel cross-sections. The ABM simulations were paused at a fixed time to update the hemodynamics in the ABM-generated vessel geometry. Ten ABM simulations were run for each plane to account for stochasticity. At the defined coupling time, plaque location, plaque size and lumen contour were retrieved as ABM outputs, and their average (among the 10 simulations) taken as reference. This procedure was repeated for each plane and the ABM configuration (among 10) with the minimum deviation from the average condition (computed for the specific plane in terms of the above-mentioned geometrical features) was used to reconstruct the resulting 3D vessel geometry. The influence of the ABM to CFD coupling time was investigated by testing three coupling schemes for 14 simulated days: a fixed coupling time of 1) 7 days and 2) 3.5 days, and 3) a variable frequency consisting in a first coupling after 7 days and then every 3.5 days. Within the simulated period, the temporal lumen area trend was not affected by the adopted coupling scheme, although for some planes the shortest coupling time allowed capturing a more frequent activation and deactivation of pathologic processes. Their ABM simulated SMC, ECM and LDL dynamics to replicate arterial wall remodeling and plaque formation and progression over time as a function of WSS, computed by the steady-state CFD simulation. Specifically, in case of at least one WSS value lower than 1 Pa (value chosen according to femoral artery data (Schlager et al., 2011)) in the considered ABM plane, an atherogenic condition was activated in the intimal layer, promoting LDL infiltration and increasing SMC proliferation/ECM production probabilities. The atherogenic threshold influence on the model output should be assessed through a robust sensitivity and uncertainty quantification analysis. Figure 6 shows relevant results of the framework along 2 months, obtained without any intermediate CFD-ABM coupling. In agreement with experimental and clinical evidence (Samady et al., 2011; Stone et al., 2012), the model simulated greater plaque formation and lumen area reduction at luminal regions exposed to low WSS. Moreover, the model successfully resembled pathological characteristics, as the development of an asymmetric plaque, characterized by the presence of a well-defined lipid core and increased intimal ECM and SMC content, coherently with experimental observations (Stary et al., 1995).

Three major differences arise with respect to the model of Bhui and Hayenga (2017). First, Glagov’s remodeling was not implemented, and, consequently, an increase in the plaque area was directly associated with lumen area reduction. Second, inflammatory cell types and cytokines as well as foam cell accumulation were not included, but a key role was attributed to SMC and ECM dynamics, neglected in Bhui and Hayenga (2017). Last, while in the work by Bhui and Hayenga (2017), plaque formation was related to WSS and LDL/leukocyte blood concentration, here only the WSS input was considered, maintaining other risk factors as intrinsic. The models by Bhui and Hayenga (2017), Corti et al. (2019) and Corti et al. (2020) did not include several underlying pathological mechanisms, such as the formation of fatty streaks, and the evolution to advanced atherosclerotic lesions with fibrous cap, necrotic core and potential calcifications. Nonetheless, both the models nicely outlined plaque formation at theoretical level and offered a solid coupling infrastructure to combine hemodynamics and cellular mechanics within the atherosclerosis development. The proposed infrastructures promise to be agile enough to serve as useful tool for clinical hypothesis testing and therapy outcome prediction, provided that an effort towards the application to realistic vessel geometries will be considered, together with a quantitative calibration and validation of the model on human data.

### Multiscale Models of In-Stent Restenosis

ISR after endovascular intervention remains a major drawback compromising the long-term outcome of the procedure (Mitra and Agrawal, 2006; Chaabane et al., 2013). ISR consists in the re-narrowing of the lumen mainly associated to an inflammatory-driven overexpressed SMC activity, as consequence of multiple, interrelated systemic, biologic and biomechanical factors (Mitra and Agrawal, 2006; Chaabane et al., 2013). Most biomechanical factors are attributable to the wall damage induced by PTA and stent deployment, and to hemodynamic alterations caused by the stent presence (Mitra and Agrawal, 2006; Koskinas et al., 2012; Chaabane et al., 2013). Both of them may promote a maladaptive healing process, involving the activation of an inflammatory response and sustained SMC synthetic and proliferative activity, potentially resulting in neointimal hyperplasia and ISR (Chaabane et al., 2013). The current knowledge of the mechanobiological processes governing ISR is still incomplete (Mitra and Agrawal, 2006; Terzian et al., 2017). Lately, many computational multiscale agent-based modeling frameworks focused on the investigation of arterial response to PTA and stent deployment to gain insights in the impact of the procedure and the stent design on the intervention outcome (Table 1, Supplementary Tables S1, S2).

**FIGURE 7:**
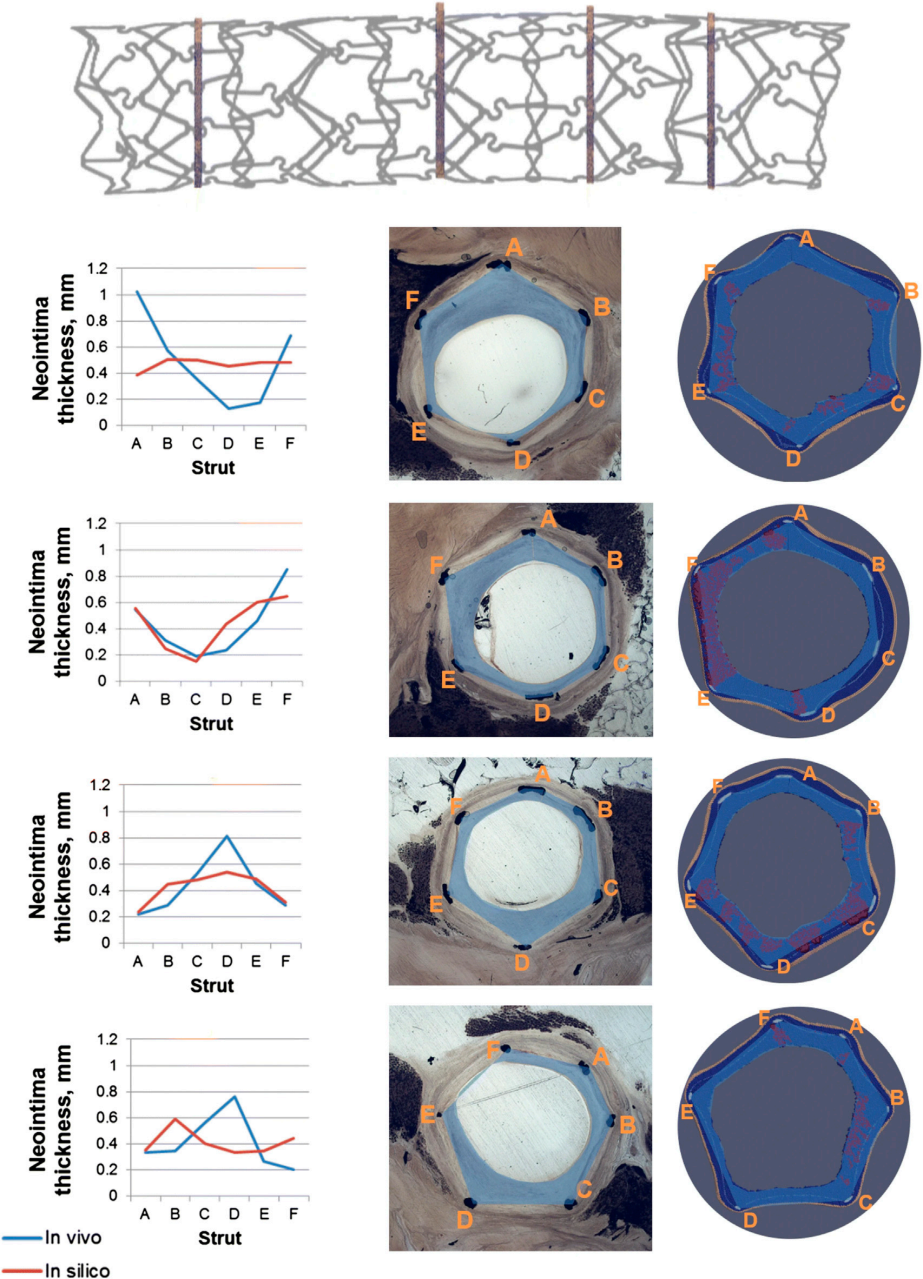
Results of the multiscale agent-based modeling framework of in-stent restenosis of Zun et al. (2019). At the top, the stent geometry is shown, with indications on the position of the four analyzed cross-sections. At the bottom, the results at 28 days are shown for the four cross-sections. For each cross-section: 1) on the left, a quantitative comparison of the predicted (*in-silico*) and *in-vivo* neointimal thickness is provided for the 6 struts locations (A–F), 2) in the middle, the *in-vivo* cross-section is shown and 3) on the right, the *in-silico* cross-section is represented, with the smooth muscle cells (SMCs) in dark blue, the internal elastic lamina (IEL) in light blue, the external elastic lamina (EEL) in beige, the extracellular matrix (ECM) in red and the stent struts in light grey. Both in the *in-vivo* and *in-silico*silico cross-sections the blue area represents the neointima estimation. Reprinted with permission from Zun et al. (2019) (http://creativecommons.org/licenses/by/4.0/).

Hoekstra’s research group proposed a modular multiscale framework to dissect the hemodynamic and mechanical effects of stenting on the pathological process of ISR and the eventual benefit of eluting anti-proliferative drugs to reduce neointimal regrowth (Caiazzo et al., 2011; Tahir et al., 2011; Tahir et al., 2013; Tahir et al., 2014; Zun et al., 2017; Zun et al., 2019). Different geometries with ascending complexity were investigated, namely 2D straight vessels (Caiazzo et al., 2011; Tahir et al., 2011; Tahir et al., 2013; Tahir et al., 2014), a 3D straight cylinder (Zun et al., 2017) and a 3D curved vessel (Zun et al., 2019). Their framework was based on the integration of four modules: 1) a Lattice-Boltzmann-based module for the computation of the hemodynamics, 2) a finite difference scheme to solve the set of PDEs for drug transport (activated only in (Caiazzo et al., 2011; Tahir et al., 2011), when the effect of drug on SMC activity was considered), 3) an ABM of tissue mechanics to compute the state of stress and strain within the arterial wall, and 4) an ABM of cellular dynamics, which replicates SMC biological activities (Caiazzo et al., 2011; Tahir et al., 2011; Tahir et al., 2013; Tahir et al., 2014; Zun et al., 2017; Zun et al., 2019). The stent deployment represents a perturbation from the model equilibrium that propagates to the other sub-modules. Specifically, the mechanical ABM simulates the stent deployment procedure, computes the resulting state of stress and determines the post-intervention configuration by removing the overstressed agents. From this initial perturbation, the ABM of cellular dynamics then regulates the mitotic activity of SMCs according to the contact inhibition criterion and in response to the mechanical, hemodynamic and drug conditions, which are updated accordingly within the fully-coupled framework. Specifically, following stent deployment and agent removal, the potential exposure of SMCs to blood flow activates the SMC mitotic phase and makes SMC activity susceptible to WSS. In the first studies (Caiazzo et al., 2011; Tahir et al., 2011), the WSS trigger was based on a simple threshold condition. In later investigations (Tahir et al., 2013; Tahir et al., 2014; Zun et al., 2017; Zun et al., 2019), the authors introduced a probability of healthy endothelium over time and a link between nitric oxide release and WSS, allowing capturing enhanced restenosis at higher stent-induced injury levels (not replicated in Tahir et al. (2011)). Further improvement saw the inclusion of the ECM, along with rules governing its production by synthetic SMCs, and a validation against experimental data of porcine coronary arteries (Zun et al., 2019). In this work, the framework was applied to stented porcine coronary arteries (idealized curved vessel with stent geometry reconstructed from micro computed tomography) and the model predictions were compared with short-term (i.e., at 14 and 28 days) histological evaluations of the same stented vessels. A good agreement between histology and simulations in terms of overall extent of neointimal thickness was obtained (Figure 7). However, some discrepancies in the local growth distribution were observed between the simulated and histological cross-sections. This was attributed by the authors to the lack of correspondence between the model geometry (based on the average characteristics of three similar porcine models) and the real *ex-vivo* vessels analyzed in the study.

Although including also the mechanical factor, the stent-derived hemodynamic-induced alteration was the main focus of the above stream of works. On a different perspective, a deep investigation of the damage induced during PTA and stenting with a multiscale agent-based modeling framework was proposed by Irish researchers (Boyle et al., 2010; Boyle et al., 2011; Zahedmanesh et al., 2014; Nolan and Lally, 2018). Their computational framework includes three modules, namely 1) a FEM module of stent deployment, 2) an ODEs module to compute the inflammatory cues and 3) an ABM module of cellular dynamics. In their original model (Boyle et al., 2010), the inflammation was triggered beyond a certain stress threshold (on the minimum principal stress) and led to the ABM initialization with growth and matrix degrading factors, assumed to constantly decrease as the cellular growth progressed. In further developments (Boyle et al., 2011; Zahedmanesh et al., 2014), two formulations of the arterial wall damage as function of the von Mises stress were proposed as either cumulative along the loading cycles (cyclic damage model) (Boyle et al., 2011) or instantaneous at the injury time (instantaneous damage model) (Zahedmanesh et al., 2014). Additionally, a more detailed model of the inflammatory variables was pursued through a set of ODEs describing the temporal variation of damage, matrix degrading and growth factors, and ECM, computed at every ABM iteration for each lattice site (Boyle et al., 2011; Zahedmanesh et al., 2014).

**FIGURE 8:**
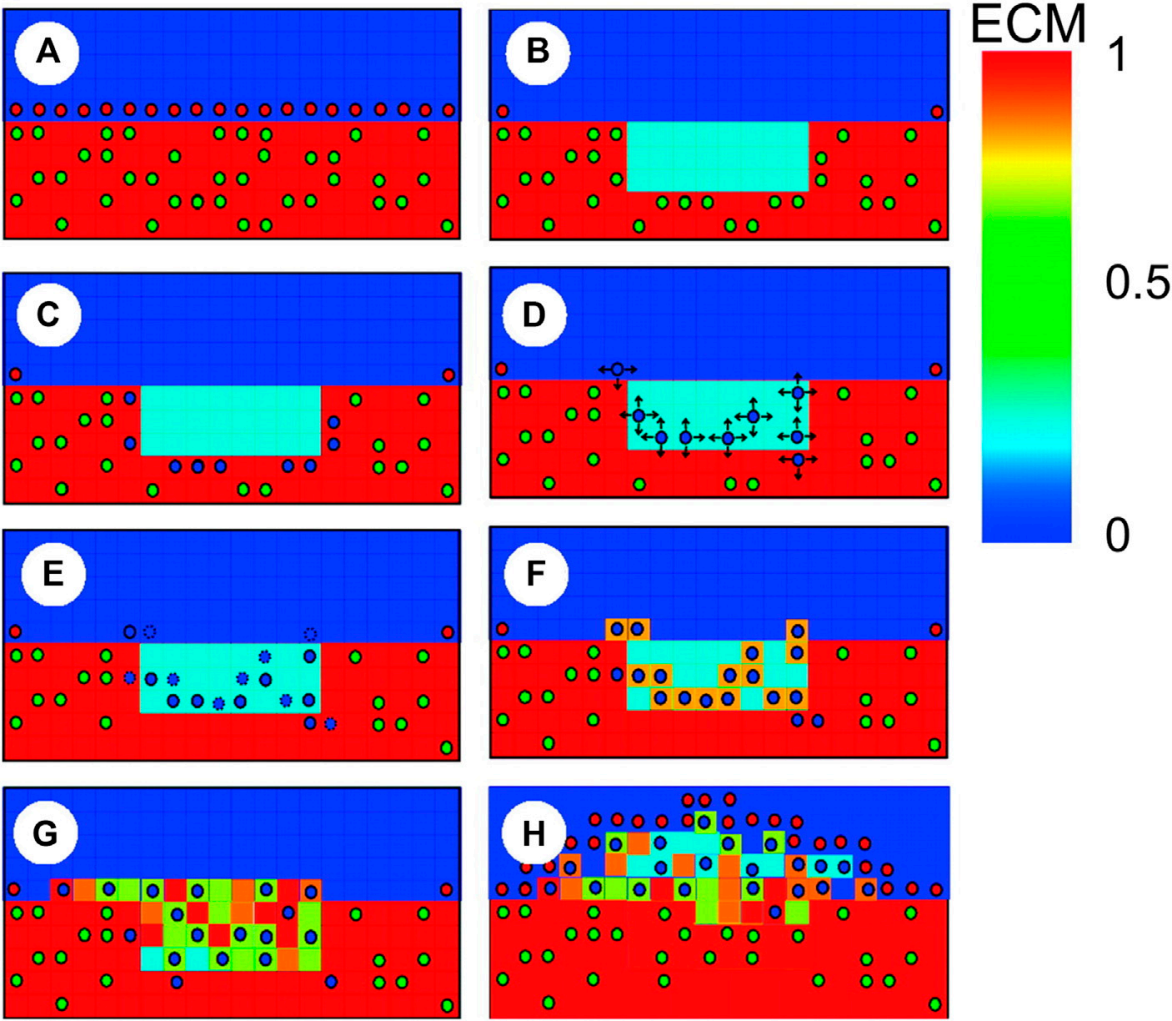
Schematic representation of agent-based model (ABM) rules in Boyle et al. (2010), with contractile smooth muscle cells (SMCs) (green circles), synthetic SMCs (blue circles) and endothelial cells (red circles). **(A)** When the endothelium and extracellular matrix (ECM) are intact, SMCs are contractile; **(B)** The injury provoked by the stent placement induces endothelial denudation, ECM reduction and SMCs removal; **(C)** In the vicinity of degraded ECM, SMCs switch to the synthetic phenotype; **(D)** Synthetic SMCs randomly migrate (arrows represent possible directions); **(E)** Synthetic SMCs proliferate (blue circles with dashed lines represent daughter cells); **(F)** SMCs produce ECM; **(G)** Lesion formation; **(H)** Reendothelialization stops lesion growth. Reprinted with permission from Boyle et al. (2010) (http://creativecommons.org/licenses/by/4.0/).

**FIGURE 9:**
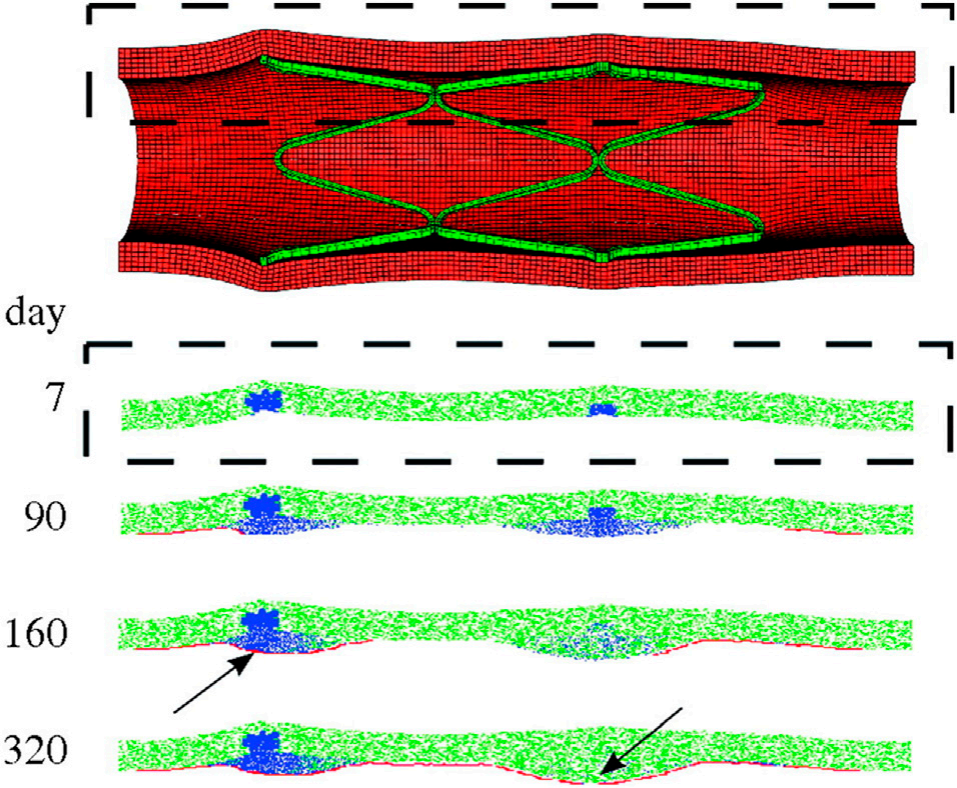
Results of the multiscale finite-element method–agent-based model (FEM-ABM) framework of in-stent restenosis by Boyle et al. (2010). At the top, the stent expansion configuration obtained from FEM analysis is shown and constitutes the initial condition of the ABM. At the bottom, the ABM evolution along 320 days is provided for a longitudinal section (dashed box). Contractile smooth muscle cells (SMCs) are represented in green, synthetic SMCs in blue and endothelial cells in red. At day 7, the ABM is characterized by a complete endothelial denudation and synthetic SMCs in the injured region (in correspondence of the stent struts). Lesion progression is shown at day 90 and 160. The endothelium starts recovering, leading to a complete reendothelialization at day 320. The lesion growth stops when complete reendothelialization occurs (arrow, day 160) or when the SMCs switch back to a contractile phenotype (arrow, day 320). Reprinted with permission from Boyle et al. (2010) (http://creativecommons.org/licenses/by/4.0/).

The ABM of cellular dynamics of the referenced works (Boyle et al., 2010; Boyle et al., 2011; Zahedmanesh et al., 2014; Nolan and Lally, 2018) was based on the same general hypotheses, although some differences in the adopted rules were introduced (Supplementary Table S2). A schematic representation of the ABM rules is shown in Figure 8. Commonly, the intervention-induced damage triggered SMCs to produce matrix degrading factors, progressively reducing the content of ECM. In case the ECM decreased below a certain value, SMCs switched to a synthetic phenotype, whose proliferation depended on the contact inhibition, growth factors (Boyle et al., 2010, 2011) and endothelial cells (when included, in Boyle et al. (2010), Zahedmanesh et al. (2014) and Nolan and Lally (2018)). In Boyle et al. (2010), Zahedmanesh et al. (2014) and Nolan and Lally (2018), a total or partial endothelial denudation was assumed in proximity to the stent struts as initial configuration (Figure 9). Then, a constant endothelial cell proliferation along the luminal surface was modeled, potentially leading to complete re-endothelialization and growth arrest (Figure 9, day 320). The endothelium recovery had an inhibitory effect on SMC activity through the release of nitric oxide. Specifically, a distance-based rule was introduced, according to which a SMC agent switched back to a contractile phenotype if an endothelial cell was present within a radius of 60 μm (Zahedmanesh et al., 2014; Nolan and Lally, 2018), determining intimal growth interruption after re-endothelialization. Additionally, when SMCs were in their synthetic phenotype, they produced ECM at constant rate and, once the ECM level reached the physiologic value, they switched back to quiescence. Finally, with the exception of the work by Nolan and Lally (2018), random migration of synthetic SMCs was implemented, regulated by the contact inhibition criterion.

The framework of Zahedmanesh et al. (2014) predicted enhanced SMC activation and intimal growth as well as delayed stabilization for stents with larger diameter or thicker struts, in agreement with the clinical evidence. Moreover, as investigated by Nolan and Lally (2018), the instantaneous damage model provided a more realistic replication of the ISR process than the cyclic damage model. Finally, consistently with *in-vivo* observations, in the instantaneous damage model a greater endothelial denudation was associated with enhanced lumen area reduction. Conversely, this key role of the endothelial injury on ISR was not captured with the cyclic damage model.

While the framework proposed by the Irish researchers (Boyle et al., 2010; Boyle et al., 2011; Zahedmanesh et al., 2014; Nolan and Lally, 2018) was based on a unidirectional coupling between the solid mechanics module and the ABM, Li et al. (2019) developed a bidirectional FEM-ABM framework in which the information of the cell-scale module was delivered to the tissue-scale module and vice-versa. The framework was implemented considering the continuous damage model proposed by Boyle et al. (2011), since it allowed accounting for time-varying stress. Differently from Nolan and Lally (2018), in this work, through a different model setting, reasonable results were obtained also with the continuous damage model. As regards the ABM, both SMCs and endothelial cells were included, and proliferation was the only simulated agent activity. SMC proliferation was governed by the cyclic damage model developed in Boyle et al. (2011), thus depending on the level of ECM, matrix degrading factors and damage. Moreover, similarly to Zahedmanesh et al. (2014) and Nolan and Lally (2018), the presence of an endothelial cell within a radius of 60 µm led to a synthetic to contractile SMC phenotypic switching. A monolayer of endothelial cells was introduced on the lumen surface and, as consequence of the stent-induced damage, endothelial cells in proximity to the stent struts were removed. Finally, endothelial cells proliferated only if they had one neighboring endothelial cell. The authors performed simulations employing both unidirectional and bidirectional coupling, with and without endothelial cells. From the results of their study, it emerged that 1) the bidirectional coupling produced a slower lumen area reduction and less dispersion among the ABM repetitions than the unidirectional one and 2) the inclusion of endothelial cells led to the suppression of SMC proliferation once the complete re-endothelialization was achieved, thus resulting in a lower lumen area reduction compared to the cases without endothelial cells. The framework was only applied to a longitudinal section of an idealized vessel geometry. Further investigation on a more realistic 3D vessel geometry is required.

### Multiscale Models of Bypass Graft Remodeling

Alternatively to endovascular procedures, bypass surgery may be preferred depending on lesion characteristics (e.g., lesion site, length, severity, calcifications) and patient-specific conditions (e.g., age, comorbidities) (Neumann et al., 2019). Bypass surgery is a revascularization procedure consisting in the anastomose of a vessel segment (either a healthy artery or vein, or an artificial graft) above and below the blocked or narrowed artery to create a parallel route for blood flow. Neointimal hyperplasia represents a critical drawback of vein graft bypass surgery affecting the long-term success of the procedure (Collins et al., 2012), for which switching from a venous to an arterial environment and the surgery associated quando mettiamo stent-induced, hemodynamic-induced… forse dovremmo mettere surgery-associated trauma play a key role (Rehfuss et al., 2018). Neointimal hyperplasia driving mechanisms in vein grafts are like those occurring in arteries undergoing endovascular intervention. Among them are the phenotypic contractile to synthetic switching of SMCs and the subsequent excessive proliferation and ECM deposition in response to the inflammatory activation (Collins et al., 2012).

A major contribution in multiscale agent-based modeling in this field was provided by Garbey’s research group (Hwang et al., 2013; Garbey et al., 2015; Garbey et al., 2017; Garbey et al., 2019) (Table 1, Supplementary Tables S1, S2). An early investigation of vein graft adaptation using ABM methodology was proposed in Hwang et al. (2013), in which the authors implemented a 1D (radial direction) and 2D (longitudinal section) ABM with rules for SMC, ECM and monocyte dynamics based and validated on experimental measurements (e.g., intimal thickness and fraction of SMCs undergoing division and apoptosis) from rabbit vein grafts under different flow conditions (Hwang et al., 2012). Through the available experimental data, agent probabilities were related to the WSS, analytically computed from the *in-vivo* measured flow rate. The model finely replicated the experimental intimal area growth over time at different WSS conditions. In this model, the multiscale component was not properly represented, because only events at the cell scale were simulated while the WSS (tissue-scale quantity) was analytically computed. In a further evolution (Garbey et al., 2015), the authors proposed a multiscale model that integrates hemodynamics and solid mechanics modules at the tissue scale and an ABM module at the cell scale. The framework was applied to a cross-section of an idealized vein graft model. The ABM module replicated SMC and ECM dynamics as function of the WSS and wall tension condition. The 2D ABM configuration was then given in input to the 2D FEM solid mechanics module, which computed the new structural equilibrium and provided the deformed geometry to the hemodynamics module and the wall tension to the ABM. Finally, through a finite volume scheme, the hemodynamics module computed the WSS in the current geometry and provided said information to the ABM. In a later work (Garbey et al., 2017), the analytical solutions of the WSS and wall tension were considered for a simplified vessel geometry, thus replacing the finite volume hemodynamics and FEM solid mechanics modules, respectively. The diffusion of a generic growth factor was solved through a finite difference scheme within the 2D ABM domain, to account for the transfer of the biomechanical inputs in the tissue. A further development was introduced in Garbey et al. (2019), which included an additional module for the replication of tissue remodeling, using an immersed boundary, continuum-based approach (Peskin, 2002). SMCs were described as particles moving in a highly viscous flow, allowing for cell-cell interactions.

**FIGURE 10:**
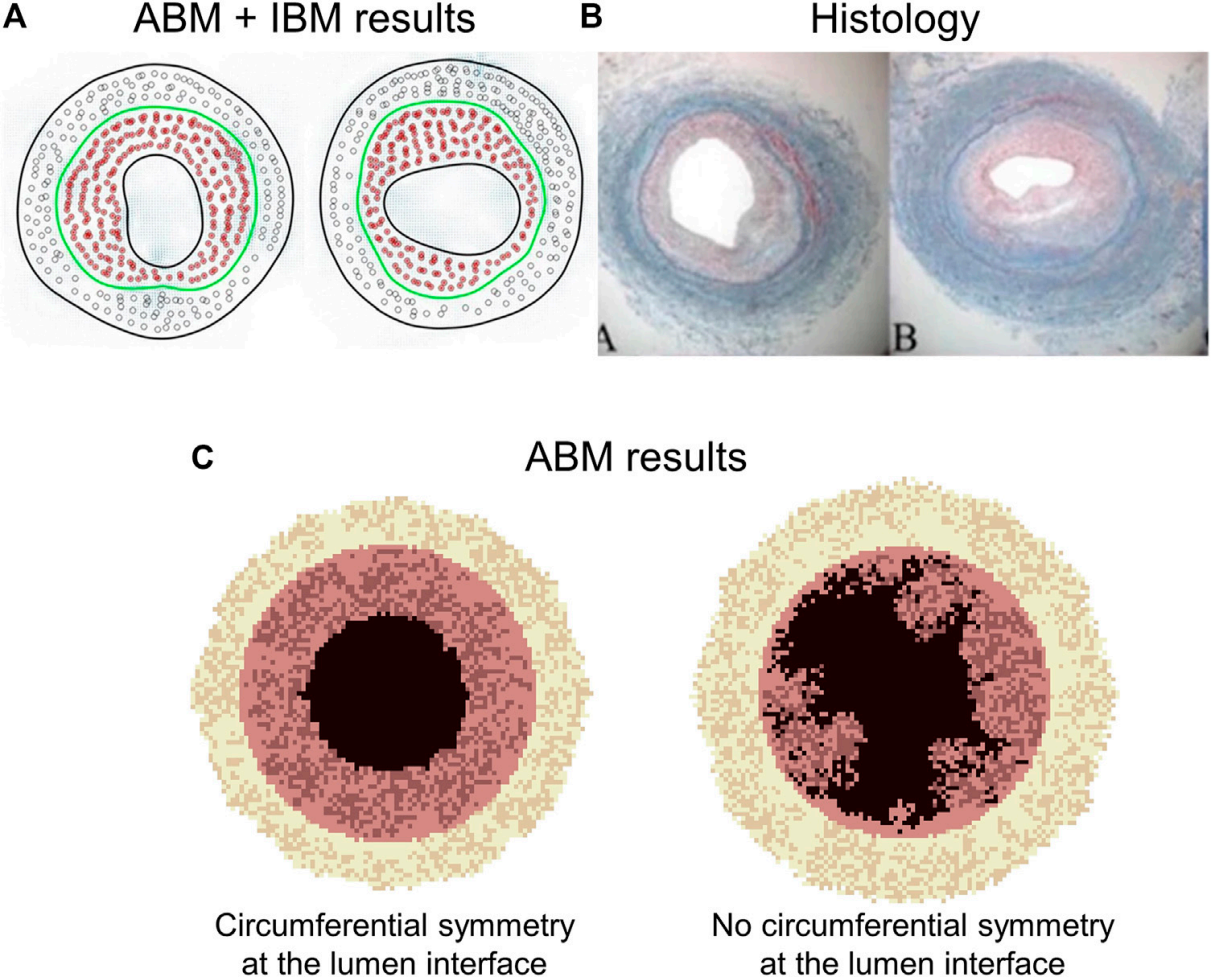
Outputs of two multiscale agent-based modeling framework of vein graft adaptation by Garbey’s research group. **(A)** Results obtained by Garbey et al. (2019), when an immersed boundary method (IBM) module was added to simulate the vessel wall remodeling. **(B)** Histological images of rabbit vein graft (Conte et al., 2006). **(C)** Results obtained by Garbey et al. (2017). The results of panel **(A)** well resemble the histological images **(B)**. The model captures different possible patterns of neointimal hyperplasia, namely a vertical and horizontal configuration (panels **(A,B)**). Panel **(C)** shows a limitation of the model by Garbey et al. (2017). In particular, when the symmetry condition is removed, a non-realistic configuration of the lumen contour is obtained (e.g., detached elements, holes). Figures 9A,B Reprinted with permission from Garbey et al. (2019) (http://creativecommons.org/licenses/by/4.0/). Figure 9C reprinted with permission from Garbey et al. (2017) (http://creativecommons.org/licenses/by/4.0/).

The common key agents for the above-described ABMs are SMCs and ECM, whose proliferation and synthesis are regulated by WSS and wall tension (Supplementary Table S2). Specifically, low WSS promoted SMC proliferation and ECM production in the intima (inward remodeling), while high wall tension promoted SMC proliferation and ECM production in the media (outward remodeling). The results obtained in terms of intimal and medial area over time, as well as SMC and ECM content temporal evolution, were consistent with experimental observations (Hwang et al., 2012; Hwang et al., 2013; Garbey et al., 2015; Garbey et al., 2017; Garbey et al., 2019) (Figures 10A,B). The ABM proposed in Garbey et al. (2017) was limited in the capability to generate lumen morphologies close to the pathophysiological reality. A smooth and regular lumen contour was retrieved only under a circular symmetry assumption (Figure 10C), while if the symmetry assumption was removed, the lumen border assumed an irregular and excessively discontinuous profile, not observed in histological images (Conte et al., 2006). This limitation was overcome introducing the immersed boundary approach (Garbey et al., 2019).

Finally, an interesting aspect highlighted by Garbey’s research group was related to the cross-validation of the agent-based modeling framework with a previously developed dynamical system, which described the same cellular events thorough a set of ODEs (Garbey and Berceli, 2013). This procedure allowed the researchers to choose one approach or the other depending on the specific purposes of the study. The ODE approach guarantees immediate computation but lacks a topological detail, while the ABM approach provides detailed morphological and compositional outputs but comes with higher computational burden.

### Multiscale Models of Other Vascular Applications

Despite the great interest in atherosclerosis and post-intervention vessel remodeling, multiscale ABMs were also proposed in other vascular arenas (Table 1, Supplementary Tables S1, S2).

A coupled FEM-ABM framework was developed by Keshavarzian et al. (2018) to simulate arterial remodeling following transient increases in blood pressure and changes in production of soluble factors (e.g., growth factors, proteases) in a 3D idealized model of porcine left anterior descending coronary artery. The model performance was assessed by evaluating both the homeostatic stability and the capability to recover transient pressure changes. In addition, the framework was applied to a model of rabbit common carotid artery to simulate the response of the vessel to the placement of a cuff. The framework was based on the bidirectional coupling between 1) a 3D FEM module, computing the stress and strain values at the tissue scale, based on the vessel morphology and composition (through a content-based strain energy function (Zulliger et al., 2004; Karšaj and Humphrey, 2012)) and 2) a 3D ABM module, replicating the cellular activities in response to the mechanical stimuli. The 3D ABM was based on a three-layered structure (i.e., intima, media and adventitia layers) and was composed of two main classes of agents, namely patch and cell agents. Each patch agent contained cells, filling ECM and soluble factors (e.g., chemokines and growth factors) and, depending on the cell-type content, it was associated to an intimal, medial, adventitial or boundary type. Endothelial cells, SMCs and fibroblasts were modeled. Agent rules were defined to replicate cell mitosis and apoptosis, production of soluble factors, and production and degradation of ECM (collagen, elastin and gelatin). Moreover, the diffusion equations in the ABM were solved through a forward in time, centered in space, discretization algorithm. The proposed framework and its application captured the chemical-cellular-tissue interplay governing vascular remodeling. First, the ability to replicate vascular homeostasis and recover from a transient 30% increase in blood pressure was verified. Then, through a sensitivity analysis, the pivotal role of collagen in stress-induced arterial remodeling emerged. Indeed, changes in the collagen mass led to modifications in the mechanical stress, in turn affecting cell and ECM dynamics. Finally, the placement of the cuff in the carotid artery model produced a decrease in the mechanical stress, leading to a decrease of SMC and collagen content, as observed in animal experiments (Bayer et al., 1999).

Zahedmanesh and Lally (2012) developed a multiscale FEM-ABM framework to investigate the remodeling mechanisms of vascular tissue-engineered scaffolds. These constructs may experience intimal hyperplasia due to an unfavorable adaptation process that results in excessive SMC synthetic activity. Mechanical factors (e.g., scaffold compliance) and loading conditions influence SMC activity by affecting the cyclic strain and the pore fluid velocity. In this context, the framework of Zahedmanesh and Lally (2012) investigated the effects of cyclic strain and pore fluid velocity, quantified through a FEM module, on SMC and ECM dynamics, simulated with a lattice-free ABM. Within an iterative approach, the FEM module transferred the mechanical inputs to the ABM, which simulated the subsequent tissue growth and remodeling and provided the new geometry and composition to the FEM module, that updated the mechanical condition accordingly. The lattice-free ABM of cellular behavior was implemented to replicate SMC migration, proliferation, apoptosis and ECM production in response to the cyclic strain and pore fluid velocity conditions. While a random migration was assumed, the rules for cell mitosis, apoptosis and ECM production were derived from experimental studies. The framework was applied to a longitudinal section of an axisymmetric geometry. As outcome, the hypertension promoted greater SMC proliferation, by reducing the cyclic strain, consistently with clinical studies reporting arterial thickening and stiffening under hypertensive conditions (London et al., 2004). Moreover, a pulsatile flow allowed for less wall thickening, less SMC proliferation, but more ECM synthesis, compared to a static condition, in agreement with *in-vitro* studies (Jeong et al., 2005). Additionally, under a physiologic pulsatile loading condition, a lower scaffold compliance (associated with lower cyclic strain) produced a greater increase of SMCs, compared to an arterial compliant scaffold, thus confirming the clinical observations (Salacinski et al., 2001). Finally, the removal of the fluid pore velocity effect in the arterial compliant scaffold under physiologic pulsatile loading led to a slower SMC growth. In all the explored scenarios, the simulated temporal trend of cell growth, characterized by a rapid increase followed by a plateau and a reduction, was consistent with *in-vitro* observations (Jeong et al., 2005). The study highlighted the potentialities of the multiscale framework in 1) investigating the isolated contributions of mechanical factors (extremely difficult to be achieved with *in-vitro* or *in-vivo* studies) and 2) indicating favorable scaffold characteristics (e.g., an arterial-like compliance) and possible loading conditions to obtain the desired cell growth.

## AGENT- VERSUS CONTINUUM-BASED MULTISCALE FRAMEWORKS: STRENGTHS AND LIMITATIONS

Works described in *Multiscale Agent-Based Modeling Frameworks of Vascular Pathophysiology* demonstrated that coupling agent- with continuum-based models allows successfully capturing biological information. The proposed frameworks used each specific model for the task it is most suitable for, thus taking advantages from its strengths and minimizing its limitations. Generally, these frameworks were based on 1) a continuum model for the molecular advection-diffusion-reaction processes, 2) a discrete (agent-based) model at the cellular level and 3) a continuum model for the tissue level mechanics (solid mechanics or hemodynamics). Moreover, for a more exhaustive vision related to the modeling of vascular adaptation, the reader should be directed also to multiscale frameworks entirely based on continuum models (which are not the object of this review), implying that also the cell scale is represented through ODE/PDE systems. Examples can be found in models of atherosclerosis (e.g., Cilla et al. (2014), Di Tomaso et al. (2015), Thon et al. (2018) and Pleouras et al. (2020)), ISR (e.g., Lally and Prendergast (2006), Escuer et al. (2019) and Maes et al. (2021)), vein graft remodeling (e.g., Budu-Grajdeanu et al. (2008) and Casarin et al. (2017)) and other vascular applications (see Humphrey (2021) for an extensive review on constrained mixture models of tissue growth and remodeling). The difference of these works with those reviewed in *Multiscale Agent-Based Modeling Frameworks of Vascular Pathophysiology* mainly regarded the representation of the cell scale (through a ODE/PDE versus ABM approach), which thus determined the nature of the multiscale framework to be either hybrid (i.e., based on the combination of continuum models with an ABM) or fully-continuum. Accordingly, this section will focus on the strengths and weaknesses of adopting agent-based versus continuum-based approaches at the cell scale, within a multiscale framework.

**FIGURE 11:**
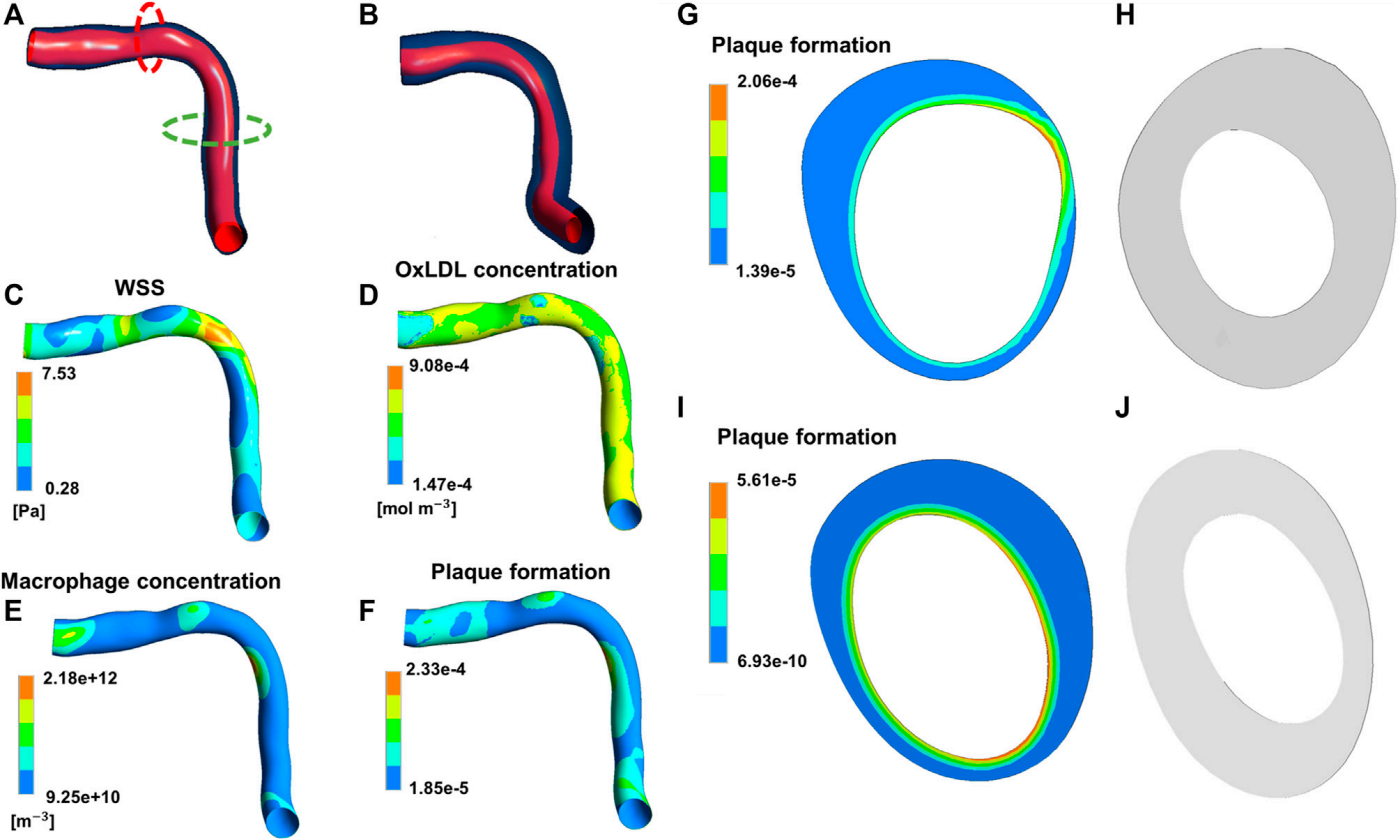
Results of the continuum-based modeling framework of atherosclerosis by Pleouras et al. (2020). Panels **(A,B)** depict the artery geometry reconstructed at the initial condition and follow-up, respectively. The contour maps of wall shear stress (WSS) **(C)**, oxidized low-density lipoproteins (LDLs) **(D)**, macrophages **(E)** and plaque formation input **(F)** at the initial condition are shown. Panels **(G,H)** refer to the red dashed line in **(A)** with the initial input of plaque formation **(G)** and the computed output cross-section **(H)**. Panels **(I,J)** refer to the green dashed line in **(A)** with the initial input of plaque formation **(I)** and the computed output cross-section **(J)**. Adapted with permission from Pleouras et al. (2020) (http://creativecommons.org/licenses/by/4.0/).

Besides the works by Casarin et al. (2017) and Maes et al. (2021), in which a set of ODEs was adopted to describe the temporal dynamics of tissue growth and remodeling, in all the other cited continuum-based studies PDE systems were implemented to capture the spatio-temporal evolution of the species of interest (e.g., growth factors, cells, ECM components, LDL), and thus the subsequent tissue remodeling, in response to fluid or mechanical stimuli. For example, in the patient-specific atherosclerosis model by Pleouras et al. (2020), CFD simulations were coupled with a PDE system describing mass transport of monocytes, LDL, and high-density lipoproteins, and inflammatory species’ dynamics in the arterial wall, ultimately leading to plaque growth over time (Figure 11). The model predictions well replicated the *in-vivo* follow-ups in terms of plaque growth and lumen area reduction (accuracy of about 80%), thus supporting the potentialities of the proposed framework. Another example is offered by the ISR model of Escuer et al. (2019), in which the initial damage stimulus induced by stenting triggered the biological response. This response was characterized by endothelial cell denudation and subsequent repopulation, and the dynamics of growth factors and matrix-degrading metalloproteinases, which in turn affected the production and degradation of ECM, with effects on SMC contractile to synthetic switching and on the following SMC activity. Tissue growth was defined as a result of the change over time of endothelial cells, SMCs and ECM. Also in this case, the model predictions in terms of percentage of stenosis were in good agreement with clinical data (Nobuyoshi et al., 1988).

The works by Pleouras et al. (2020) and Escuer et al. (2019) provide good examples of how continuum-based frameworks offer an alternative approach to model vascular adaptation processes, with differences respect to agent-based frameworks both in modeling perspectives (top-down versus bottom-up approach) and in the obtained results (in terms of level of information), as discussed below. About basic modeling perspective, the systems behavior 1) emerges from the simulation of individual components’ behaviors and interactions in ABMs (bottom-up approach), and 2) is described through aggregate differential equations governing the population average behavior in PDE systems (top-down approach). In principle, since the population dynamics of cells and ECM derive from the behavior of each entity, the PDEs should represent the collective behavior emerging from the ABM. However, ABMs provide a deeper level of detail, beyond the aggregate properties of the system. Accordingly, as also observed from the comparison of Figure 11 with Figures 5–10, spatial compositional heterogeneity and morphological-related features (e.g., tissue composition and distribution of cells and ECM or growth shapes and lumen irregularities) are more naturally captured in ABMs than in PDE systems (even when constrained mixture models are considered).

Moreover, in ABMs, cellular activities are often modeled through “if-then” rules to describe different behaviors the agents assume according to the specific scenario. This aspect was not included in the aforementioned frameworks based on PDEs. Indeed, although PDEs can embed discontinuous behaviors through properly defined constraints, the individual nonlinearities are more naturally captured through ABM rules (Bonabeau, 2002). Generally, the set of cellular behaviors described by ABMs easily span from a system of few simple rules, in which only the key activities are simulated, as cell mitosis and apoptosis, and ECM production and degradation (Garbey et al., 2019; Corti et al., 2020), to numerous and complex activities accounting for production of specific molecules and interaction between cells (e.g., contact inhibition criterion or the silencing effect of endothelial cells on SMC proliferation (Zahedmanesh et al., 2014)). Since ABMs describe phenomena from the perspective of the active component, adding a behavior to the agent implies defining a new rule, without changing the basic set of rules. ABMs can indeed replicate complex systems through a stepwise process. Conversely, PDE systems become cumbersome when an elevated number of equations is included, and the inclusion of new equations is not as easy and intuitive as in ABMs.

**FIGURE 12:**
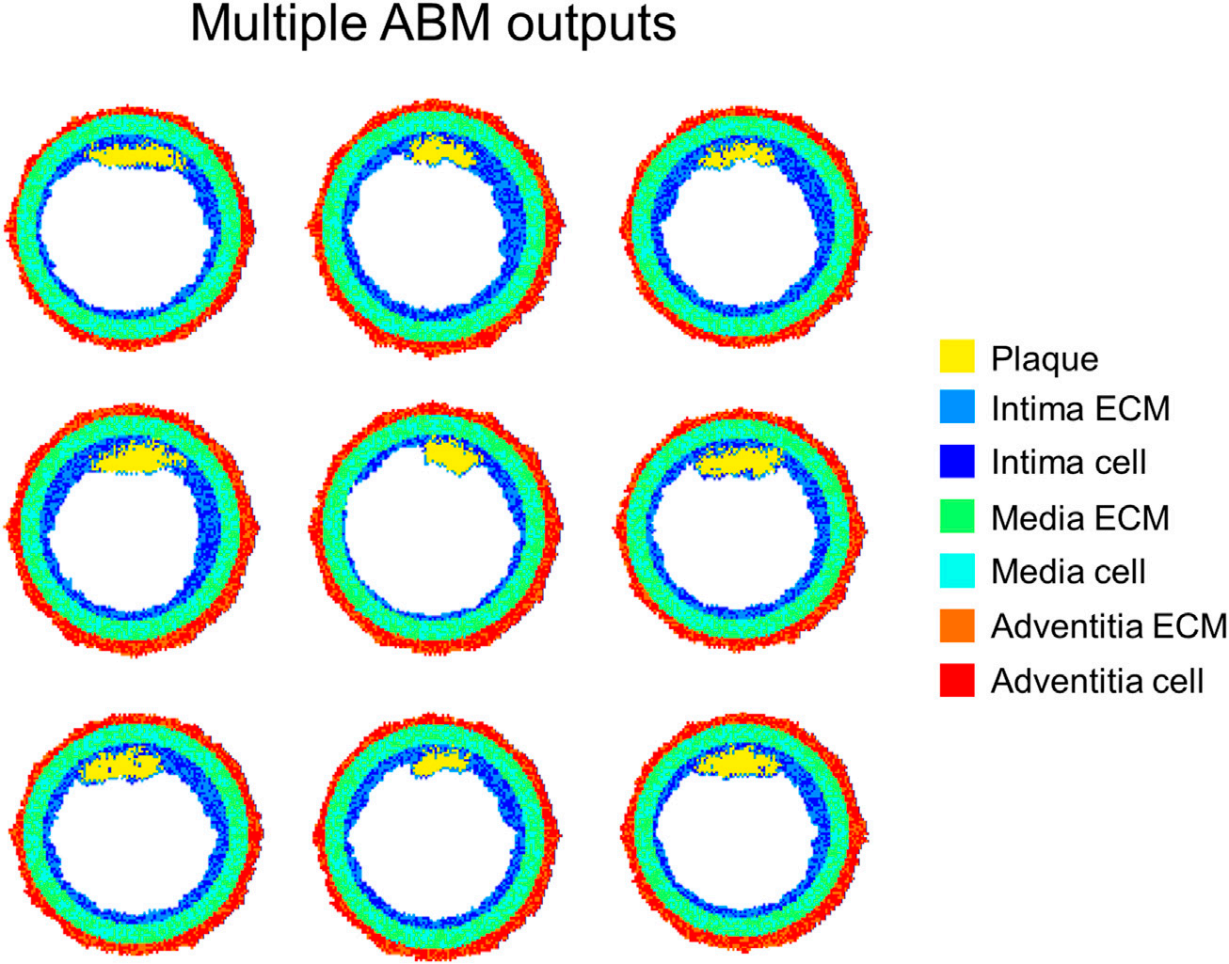
Agent-based model (ABM) outputs at 2-months follow-up obtained from 9 independent simulations of the same ABM cross-section initialized with equal WSS profile. Figure inspired from the work of Corti et al. (2020).

Additionally, stochastic ABMs and deterministic continuum models are usually developed, as also reflected by the studies previously reviewed. The stochasticity in ABMs is often introduced by defining probabilistic behavioral rules. This allows embedding a certain degree of randomness resulting in the generation of multiple possible evolution outputs of the system from a given initial condition. Conversely, the implementation of stochastic differential equations is less common, although possible (Székely and Burrage, 2014), and, to the best of the authors’ knowledge, it was never applied to the modeling of vascular adaptation. In all the continuum-based modeling frameworks, deterministic differential equations were implemented, and a unique solution of the system was produced, in agreement with the average of the observations of the specific phenomenon. Differently, the stochastic agent-based modeling frameworks reviewed in *Multiscale Agent-Based Modeling Frameworks of Vascular Pathophysiology* generated multiple solutions, thus reflecting a realistic scenario in which a population of observations is usually obtained in biology-related contexts (i.e., *in-vitro* or *in-vivo* animal and human studies). An example of multiple outputs obtained from an agent-based modeling framework of atherosclerosis is depicted in Figure 12. The figure shows 9 ABM outputs of compositional and morphological evolution of an idealized femoral artery cross-section, being exposed to an atherogenic CFD-derived WSS profile. All the configurations present similar stenosis degree as well as plaque size and location. However, they express an intrinsic variability due to the model stochasticity, consistent with biological systems.

ABMs to simulate cellular behavior present some weaknesses. The major limitation is related to the computational costs associated with a discrete stochastic model (see also *Computational Speed-Up*). The simulation of each agent dynamics is computationally more expensive compared to that of the aggregate population through PDE systems. ABM simulations require hours up to days, while ODE/PDE-based simulations typically run in the order of seconds. For instance, in the work of Garbey et al. (2017), the re-vascularization dynamics of a vein graft was simulated for a follow-up period of 6 months with both an ABM and a dynamical system (ODE-based). The computational burden was of 24 h (ABM) versus few seconds (ODE). Additionally, ABM stochasticity imposes multiple runs to reproduce the full population distribution and thus to obtain a result comparable with the available data. Conversely, deterministic models provide a unique solution, which generally replicates a statistically meaningful representation of the system trend (Székely and Burrage, 2014). Consequently, the computational power required by discrete and stochastic models might limit the spatial dimension of the model and the temporal window of the simulated processes.

In summary, the integration of agent- and continuum-based approaches in a multiscale agent-based modeling framework constituted a successful choice to model vascular adaptation, allowing 1) exploring the mechanobiological processes at a deeper level of details, highlighting the importance of spatial heterogeneity and local morphological peculiarities, 2) capturing emergent properties of the system and 3) including randomness. Moreover, such a hybrid scheme exalted the potentialities of continuum models at the molecular and tissue scales and of ABM at the cell scale. Indeed, 1) PDEs are ideal for modeling the spatio-temporal evolution of concentration profiles of molecules that are transported through diffusion and/or convection in the tissue and are subjected to well-defined reactions, 2) ABMs efficiently describe “active” entities, as cells that proliferate, die or produce and degrade ECM and 3) continuum models are typically adopted at the tissue scale to model solid mechanics and hemodynamics, generally through FEM or finite volume method.

## Challenges and Future Directions

### Verification, Uncertainty Quantification, Calibration and Validation

The advent of computational modeling in vascular pathologies (and other) have fostered a deep discussion on how much we can rely on the simulation outcome (Viceconti et al., 2021). Recently, the American Society of Mechanical Engineers (ASME) published a technical standard for the assessment of the computational model credibility in the context of medical devices, specifying the requirements in terms of context of use of the model (i.e., the specific role and scope of the model), risk assessment, verification, validation and uncertainty quantification (ASME, 2018). Consequently, the model credibility is obtained for the declared context of use. Similar concepts were also reported in the European Medicines Agency (EMA) guideline for the qualification of pharmacokinetic models and simulations (EMA, 2018). These guidelines only refer to medical devices and pharmacokinetics, thus future efforts are needed to define suitable protocols and methods for wider biomedical applications. The criticality of computational model verification, uncertainty quantification, calibration and validation in the biomedical field is also demonstrated by recent publications (Marino et al., 2008; Luraghi et al., 2018; Nikishova et al., 2018, 2019; Fleeter et al., 2020; Ye et al., 2021a; Curreli et al., 2021; Groen et al., 2021; Luraghi et al., 2021; Rapadamnaba et al., 2021).

Model verification consists in the demonstration that the computational model behaves as expected from the mathematical formulation, implying that there are not implementation errors, the equations are correctly solved and the introduced numerical errors do not significantly affect the solution (ASME, 2018). Validation confirms that the computational results well replicate the experimental observations, and the model can reliably simulate the real phenomenon (ASME, 2018). Finally, uncertainty quantification analysis deals with 1) the measurement of the model uncertainty in the output, related to the uncertainties in the input parameters (epistemic uncertainty) or to the model stochasticity (aleatory uncertainty), and 2) the investigation of how the parameter variations affect the model output, known as sensitivity analysis (ASME, 2018; Ye et al., 2021b). The uncertainty and sensitivity analyses can be performed either at early stages of model development or at the end of the validation process. In the first case (i.e., early development), uncertainty and sensitivity analyses are useful instruments to gain insights into the functioning of the model (e.g., how the model responds to variations in the inputs) and to identify which are the most influencing model parameters, whose accurate definition would allow improving the model prediction (parameters associated with high uncertainty in the model output, and whose variation determines a large oscillation of the model response), as done for instance in Corti et al. (2020). In this context, uncertainty and sensitivity analyses can be also preliminary to model calibration, the process through which the model parameters are tuned to fit experimental data. This is particularly useful if many model parameters must be calibrated, so that first attention might be paid to the most influencing parameters identified through uncertainty and sensitivity analyses. In the second case (i.e., after the validation process), an additional measure of the reliability of the computational model would be provided. Indeed, the model might agree with experimental data, meaning that it is validated, but, at the same time it might have low credibility, due the large uncertainty associated with the output (Viceconti et al., 2021).

All these processes are challenging, yet fundamental phases of the modeling activity. When dealing with multiscale frameworks integrating individual sub-models, it is good practice to verify, analyze and validate each module first, and then proceed with the whole multiscale framework (Walpole et al., 2013). This increases the computational efforts to achieve the model credibility compared to those required in case of an individual, single-scale model. Moreover, multiscale agent-based modeling frameworks present more issues compared to their deterministic and fully-continuum counterparts.

A strict definition of what the ABM verification implies, and which are the suitable methods for this purpose, is lacking. While verification methods for deterministic and continuum models (based on ODE/PDE systems) are well documented, the literature lacks rigorous methods for ABMs. A rare example of model verification workflow for ABMs was proposed by Curreli et al. (2021), and applied to a stochastic ABM of Mycobacterium tuberculosis infection. Both deterministic and stochastic model verifications were performed. In the first case, the random variables were fixed and 1) the existence and uniqueness of the solution, 2) the temporal discretization errors, 3) the smoothness of the solution and 4) the model outputs at different parameter sets were evaluated. In the second case, the random variables were “activated”, and the input parameters were fixed, thus the robustness of the model at multiple runs was assessed and the minimum number of simulation repetitions needed to achieve statistical significance was computed.

Uncertainty and sensitivity analyses require an elevated number of independent simulations to obtain a good estimation of the uncertainties and to capture significant correlations between input parameters and model outputs. In addition, if the model is stochastic, characteristic of all the multiscale agent-based modeling framework reviewed in *Multiscale Agent-Based Modeling Frameworks of Vascular Pathophysiology*, a certain number of repetitions must be performed to account for the aleatory uncertainty, typically as many as to bring the standard deviation to a stable plateau. Consequently, these tasks may become extremely time-consuming and almost unfeasible. This roadblock can be addressed by either employing ABM-suitable computational languages that speed up the simulations, or by resorting to surrogate models (or metamodels) that drastically reduce the model complexity, as discussed in *Computational Speed-Up*. Most of the studies reviewed in *Multiscale Agent-Based Modeling Frameworks of Vascular Pathophysiology* analyzed the response of the model to the variation of certain parameters (e.g., stent deployment depth, stent strut dimension, endothelial recovery rate) to explore the model behavior under specific conditions, which can be easily linked to meaningful considerations from a clinical/biological viewpoint. For example, tuning the stent-related parameters highlighted the potentiality of the model in providing a tool for testing the arterial response to different stent designs. However, a robust uncertainty quantification or sensitivity analysis is generally lacking. Some contribution in this context derived from Hoekstra’s research group (Nikishova et al., 2018; Nikishova et al., 2019; Ye et al., 2021a), which proposed a workflow for the uncertainty quantification of a multiscale agent-based modeling framework of ISR. The authors stressed the high computational effort needed for these analyses if Monte Carlo methods are adopted and proposed developing surrogate models either for a sub-module (Nikishova et al., 2019) or the entire framework (Ye et al., 2021a). Both approaches were up to 7-fold faster than the Monte Carlo method and provided acceptable estimates of the uncertainties, thus demonstrating their validity and potentiality in case of computationally intensive analyses.

Uncertainty and sensitivity analyses can also be performed to identify the most influencing parameters that drive the model response, whose accurate estimation would result in a great improvement of the model prediction reliability and associated uncertainty reduction (Corti et al., 2020). Indeed, ABMs often depend on many parameters, and their calibration in a single-step process may result ineffective, especially if it is based on the evaluation of few outputs. For example, if the available patient data is solely the lumen area over time (as generally occurs), the calibration of many parameters in a single step may not be the optimal choice. The calibration problem may be reduced to only those parameters that are strongly associated with the output of interest (i.e., the lumen area), achieving a good compromise between computational efforts and model accuracy. These considerations highlight the limited availability of patient data, which makes both the model calibration and the subsequent validation challenging, in particular when dealing with patient-specific models. Indeed, to validate the model, a set of patient data, different from the one used for the calibration, is necessary to demonstrate that the model predictions agree with the observations and thus that it can be reliably used for the purpose it was designed (e.g., predicting the vascular adaptation following intervention or a specific therapy). The calibration and validation of idealized models is less challenging than that of patient-specific models because suitable comparable data can be more easily obtained from *in-vitro* or *in-vivo* experiments. However, 1) experimental data used for model calibration and validation are often obtained from retrospective analyses of experiments that were not specifically designed to support the computational modeling process, and 2) most of the *in-vitro* studies refer to normal cells and not pathological ones. Consequently, also for idealized models, the availability of the data required to calibrate and validate the model is not granted. The advantage of the ABMs reviewed herein is that, since they replicate cellular behavior under specific conditions, they can be more easily related to *in-vitro* or *in-vivo* experiments. In this context, the work by Casarin et al. (2018) offered an example of how their ABM of vein graft adaptation could be used to wisely plan clinical experiments for retrieving the parameters needed to optimize the model setup.

In summary, the field of multiscale agent-based modeling of vascular adaptation still presents challenges in the area of verification, uncertainty quantification, calibration and validation that need to be fully addressed. Although these processes are fundamental for the achievement of model credibility and its potential application in the pre-clinical or clinical decisional phase, they have been poorly explored for multiscale agent-based modeling frameworks. Future works in this area will be of great impact, since they will add value to this promising approach for the study of vascular adaptation.

### Computational Speed-Up

A major limitation of multiscale agent-based modeling frameworks is the high computational demand required by ABM simulations. ABMs are generally based on *for* and *while* loops that scan the entire grid and evaluate each agent dynamics in response to environmental conditions and mutual interactions with other agents. Programming languages widely used in academia, such as Matlab (MathWorks), NetLogo (http://ccl.northwestern.edu/netlogo/, (Wilensky, 1999)), Repast (http://repast.sourceforge.net/, (North et al., 2013)) etc., offer great visualization tools that allow for an easier model development and testing but fail on execution speed. Languages such as C/C++ or Java are way more suitable to solve complex models laying on nested *for* and *while* loops. However, they are also not always user-friendly for computational biologists and they are poor in visualization tools. Matlab has available a mid-way solution in the form of the coder toolbox that allows “translating” a code developed in Matlab into C language. The toolbox has an intuitive interface and provides a remarkable gain in the computational runtime (Casarin et al., 2018; Dondossola et al., 2019). Nevertheless, many pre-implemented Matlab functions are not available in C language, forcing the developer to build his own C-compatible function.

Surrogate models mimic the behavior of the original computational model, by providing an estimation of the outputs of interest, while getting rid of the original model complexity and being computationally cheaper. They behave as a black-box replicating only the input-output response of the original model, without any detail of the inner system dynamics and working mechanisms. Once validated, the surrogate model can replace the original one thus allowing performing a huge number of simulations at a lower computational cost. This is useful in tasks that require the collection of an elevated quantity of outputs or model evaluations, as in sensitivity analysis, uncertainty quantification, or model calibration. An example of the latter is offered by the work of Casarin and Dondossola (2020) through the integration of machine learning-based random forest algorithm in the pipeline of ABM calibration. Here, a deep learning algorithm was fed with a certain (usually large) number of ABM-generated data points to “learn” the intrinsic model dynamics that depended on the unknown coefficients (data-driven approach). The output of the surrogate model was then compared with experimental data of reference and their difference minimized with a genetic algorithm.

Other research groups focused more on code parallelization combined with the use of supercomputers with a huge number of central processing units (CPUs) and/or graphics processing units (GPUs). In this direction, Randles et al. (2021) used a large-scale supercomputer to run their ABM (36 million CPU hours on 131,072 cores). Although extraordinary, it is clear how this approach is only feasible when massive, optimized parallel computing resources are available.

### Modeling of Molecular Pathways

In the emerging field of personalized medicine, the so-called omics sciences, including genomics, proteomics, transcriptomics and metabolomics, are recently receiving great interest. The omics data allow identifying patient-specific pathophysiological pathways, thus providing insights into the patient’s disease mechanisms, and potentially leading to the development of tailored therapies. The integration of multi-omics data in multiscale models of vascular adaptation is thought to provide a remarkable contribution in the understanding of cardiovascular diseases (e.g., through the discovering of disease biomarkers) and, as consequence, in the disease prevention, diagnosis and treatment (e.g., pharmacogenomics and pharmacoproteomics) (Ouzounian et al., 2007). For instance, a gene expression network can be included to explicitly model the intracellular signaling pathways and its effect on cellular activities and tissue remodeling. Consequently, the vascular adaptation process resulting from the up- or down-regulation of specific genes may be predicted through a multiscale framework involving the gene, molecular, cell and tissue scales. This was done by Casarin et al. (2017), who proposed a fully-continuum multiscale framework of vein graft adaptation, based on the following two modules: 1) a system of ODEs replicating gene expression dynamics and 2) a system of ODEs describing the temporal dynamics of SMCs and ECM as function of gene expression and WSS. The framework was calibrated on experimental data (i.e., histomorphology measurements, gene expression, flow rate measurements) obtained from a rabbit model of bilateral vein graft. The proposed framework can be used to explore the impact of specific perturbations of gene dynamics on the following vein graft adaptation, thus providing a virtual platform to identify gene therapeutic targets, whose manipulation would promote a successful vein graft outcome. This constitutes a step forward towards the future of personalized medicine.

Although continuum models of gene-protein networks were successfully combined with ABMs of cellular behavior in the context of cancer modeling (Mansury and Deisboeck, 2004; Zhang et al., 2007; Zhang et al., 2009a; Zhang et al., 2009b), to the best of the authors’ knowledge, similar approaches have not been proposed in vascular adaptation yet. Such hybrid models would promise to make a significant impact in vascular disease drug development and therapy optimization. After all, most of molecular therapy advancements today either originate from already existing approaches (being so most of the time only incremental), or they emerge unexpectedly from studies with a different objective, or they arise from processes that are decades-long, incredibly expensive and with no guarantee of success. Accordingly, considering 1) the relevant findings of the continuum gene-cellular framework of Casarin et al. (2017) and 2) the successful application of multiscale agent-based modeling frameworks integrating multi-omics data in the cancer research field, the authors’ opinion is that the development of multiscale agent-based modeling frameworks of vascular adaptation including gene or protein networks would be an extremely interesting research area to be explored in the near future.

## Conclusion

In this review, the state-of-the-art of computational multiscale agent-based modeling frameworks of vascular adaptation was presented, demonstrating that coupling continuum- with agent-based models is a successful approach for simulating the behavior of complex biological systems, and especially for capturing the mechanobiological mechanisms underlying vascular response to biological, chemical and mechanical stimuli. First, a multiscale model is deemed fundamental, being the nature of the system inherently multiscale: the tissue/organ response is just the tip of the iceberg, resulting from the complex network of interactions across different spatio-temporal scales. Then, each scale presents peculiar features, making it more suitable for either a continuum or discrete model. Specifically, while the extracellular molecular transport and the solid mechanics or hemodynamics at the tissue scale are well described by continuum models (ODE/PDE systems), cellular behaviors are more naturally and effectively replicated by ABMs, which, through a bottom-up and systems biology approach, allow capturing the emergent behavior of the system arising from the action and interaction of individual entities (e.g., cells).

To the authors’ opinion, the inclusion of cell-scale ABMs in a multiscale framework of vascular adaptation, compared to fully-continuum frameworks, adds value to the description of the biological system by providing greater details on morphological-related features, tissue heterogeneity and by capturing the intrinsic randomness. However, this approach is not without limitations, which are mainly related to the high computational costs, and challenges, as those regarding the processes of verification, uncertainty quantification, sensitivity analysis, calibration and validation, for which robust and efficient methods need to be developed. In fact, most of the studies presented herein provided sophisticated methodologies to model vascular adaptation processes but lacked in calibration and validation. The assessment of the credibility of these models is an essential requirement that should be addressed before they can be used as a practical tool for the improvement of current therapeutical approaches in vascular medicine and the development of new ones. For example, the computational frameworks discussed herein might be used to test drugs acting on specific pathological processes, or different stent designs (e.g., strut thickness, shape) or deployment procedure (e.g., deployment depth) either on idealized cases, as a preliminary study to exclude the worst solutions and drive further experimental research on the most promising ones, or on patient-specific cases, to optimize the personalized therapy.

Finally, the present review, by addressing the state-of-the-art of multiscale agent-based modeling frameworks of vascular mechanobiological processes, aimed to inspire researchers for future investigations of novel and unexplored scenarios within the cardiovascular field. Multi-omics data, defining patients’ molecular signature, were never explicitly included in multiscale agent-based model frameworks of vascular pathophysiology. The integration of these data into the models could markedly increase the understanding of vascular diseases and improve the diagnosis, prognosis and treatment in the context of personalized medicine, which is expected to revolutionize the approach to cardiovascular diseases in the near future.
