# Supplementary material for: Multiscale Computational Modeling of Vascular Adaptation: A Systems Biology Approach Using Agent-Based Models
Source: Front Bioeng Biotechnol. 2021 Nov 2;9:744560. doi: 10.3389/fbioe.2021.744560 (PMC8593007; doi:10.3389/fbioe.2021.744560)
Supplement: Supplementary file 1 [file Table1.pdf]

frontiers

Supplementary Material

1 Supplementary Tables

Supplementary Table S1 – Multiscale agent-based modelling frameworks of vascular adaptation: details on the modules and software.

Authors (Year)	Pathology	Module integration	Software
Bhui and Hayenga (2017)	Atherosclerosis	Fully-coupled framework between hemodynamics module (tissue scale) and ABM module (cell scale)	Hemodynamics module: COMSOL<br>ABM: NetLogo 3D<br>Coupling: Matlab
Corti et al. (2019),<br>Corti et al. (2020).	Atherosclerosis	Fully-coupled framework between hemodynamics module (tissue scale) and ABM module (cell scale)	Hemodynamics module: Fluent<br>ABM: Matlab<br>Coupling: Matlab
Caiazzo et al. (2011),<br>Tahir et al. (2011),<br>Tahir et al. (2013),<br>Tahir et al. (2014),<br>Zun et al. (2017),<br>Zun et al. (2019).	In-stent restenosis	Fully-coupled framework<br>Initial condition: ABM - physical solver computes stent deployment<br>Hemodynamics module (tissue scale), ABM – physical solver (cell scale) and drug diffusion module (molecular scale) compute the input for the ABM – biological solver (cell scale).<br>ABM – biological solver passes the updated vessel geometry to the other modules.	COAST Multiscale Coupling Library and Environment (MUSCLE) – CxA dedicated software environment.
Boyle et al. (2010)	In-stent restenosis	Unidirectional coupling between the solid mechanics module (tissue scale) and the ABM module (cell scale)	Solid mechanics module: ABAQUS<br>ABM: NA
Boyle et al. (2011)	In-stent restenosis	Unidirectional coupling from solid mechanics module (tissue scale) and inflammation module (molecular scale)<br><br>Fully-coupled framework between inflammation module (molecular scale) and ABM module (cell scale)	Solid mechanics module: ABAQUS<br>ABM: Visualization toolkit and Mechanobiology ToolKit<br>Inflammation module: Visualization toolkit and Mechanobiology ToolKit
Zahedmanesh et al. (2014)	In-stent restenosis	Unidirectional coupling between the solid mechanics module (tissue scale) and the ABM module (cell scale)	Solid mechanics module: ABAQUS<br>ABM: BREVE (STEVE language)<br>Coupling: Python routine

Nolan and Lally (2018)	In-stent restenosis	Unidirectional coupling from solid mechanics module (tissue scale) and inflammation module (molecular scale)<br><br>Fully-coupled framework between inflammation module (molecular scale) and ABM module (cell scale)	Solid mechanics module: ABAQUS<br>ABM: Matlab
Li et al. (2019)	In-stent restenosis	Fully-coupled framework between solid mechanics module (tissue scale) inflammation module (molecular scale) and ABM module (cell scale)	Solid mechanics module: ABAQUS<br>ABM: Matlab
Garbey et al. (2015)	Vein graft remodeling	Fully-coupled framework between solid mechanics module (tissue scale), hemodynamics module (tissue scale) and ABM module (cell scale)	Hemodynamics module: NA<br>Solid mechanics module: FeBio<br>ABM: Matlab
Garbey et al. (2017)	Vein graft remodeling	WSS and wall tension updated at each ABM time step, within the ABM	ABM: Matlab
Garbey et al. (2019)	Vein graft remodeling	WSS and wall tension updated at each ABM time step, within the ABM	ABM: Matlab<br>IBM: Matlab
Zahedmanesh et al. (2012)	Remodeling of a vascular tissue-engineered scaffold	Fully-coupled framework between solid mechanics module (tissue scale) and ABM module (cell scale)	Solid mechanics module: ABAQUS<br>ABM: BREVE (STEVE language)<br>Coupling: Python routine
Keshavarzian et al. (2018)	Arterial growth and remodeling under different conditions: growth factors, chemicals, blood pressure	Fully-coupled framework between solid mechanics module (tissue scale) and ABM module (cell scale)	Solid mechanics module: ANSYS<br>ABM: Netlogo<br>Coupling: Java subroutine

ABM: agent-based model; FEM: finite element method; FVM: finite volume method; IBM: immersed boundary method. NA: not available; ABAQUS: Dassault Systèmes Simulia Corp., USA; Fluent: Ansys Inc., Canonsburg, PA, USA; Matlab: MathWorks, Natick, MA, USA

Supplementary Table S2 – Multiscale agent-based modelling frameworks of vascular adaptation: details about the ABM.

Authors (Year)	Pathology	Compartments	Agents	Rules
Bhui and Hayenga (2017)	Atherosclerosis	Endothelium, arterial wall (single compartment)	EC, SMC (inert agents), Leukocytes (Neutrophils, monocytes, macrophages foam cells and lymphocytes)	- Production of cytokines in the artery wall by leukocytes and migration through Fick's law<br>- LDL: from the lumen to the arterial wall as function of WSS and diffusion in the arterial wall through Fick's law. Oxidation of LDL and phagocytosis by monocytes derived macrophages, forming foam cells<br>- Leukocyte adhesion as function of cytokines and WSS<br>- Leukocyte transendothelial migration as function of stiffness<br>- Leukocyte migration guided by chemotaxis<br>- Glagov's remodeling
Corti et al. (2019), Corti et al. (2020).	Atherosclerosis	Intima, media and adventitia layers	SMC, ECM (collagen, elastin), LDL, Fibroblasts	Intima:<br>- SMCs: proliferation as function of WSS, constant apoptosis<br>- ECM: production as function of WSS, constant degradation<br>- LDL: accumulation in the intima as function of WSS<br>Media and adventitia:<br>- Constant SMC proliferation and apoptosis<br>- Constant fibroblast proliferation and apoptosis<br>- Constant ECM production and degradation
Caiazzo et al. (2011), Tahir et al. (2011)	In-stent restenosis	Media layer covered by IEL	SMC, IEL	Physical solver:<br>- Computation of the new equilibrium position of each SMC based on forces (attractive/repulsive forces, viscous friction, elastic, boundary and motility forces)<br>Biological solver:<br>- Loss of CI activates SMC mitotic state. When the stent is deployed, IEL agents are removed near the stent struts due to high stress. Consequently, neighboring SMCs enters the synthetic phase<br>- Drug>threshold inhibits mitosis<br>- WSS<threshold or OSI>threshold or Stress>threshold induce mitosis if allowed by CI and drug concentration.
Tahir et al. (2013), Tahir et al. (2014), Zun et al. (2017).	In-stent restenosis	Media layer covered by IEL (Tahir et al. 2013, Tahir et al. 2014) and EEL (Zun et al. 2017)	SMC, IEL (Tahir et al. 2013, Tahir et al. 2014) SMC, IEL, EEL (Zun et al. 2017)	Physical solver:<br>- Computation of the new equilibrium position of each SMC based on forces (attractive/repulsive forces, viscous friction, elastic, boundary and motility forces)<br>Biological solver:<br>- Loss of CI activates SMC mitotic state. When the stent is deployed, IEL agents are removed near the stent struts due to high stress. Consequently, neighboring SMCs enters the synthetic phase<br>- NO<threshold induces proliferation; NO>threshold stops proliferation<br>- NO concentration is computed based on WSS and on the presence of functional endothelium. Assumption of complete endothelium denudation after PTA and presence of functional endothelium over time after stenting from literature<br>- Scenarios of re-endothelialization either from the boundaries of the stented region or random

Zun et al. (2019)	In-stent restenosis	Media layer covered by IEL and EEL	SMC, ECM, IEL, EEL	Physical solver:<br>- Computation of the new equilibrium position of each SMC based on forces (attractive/repulsive forces, viscous friction, elastic, boundary and motility forces)<br>Biological solver:<br>- Loss of CI activates SMC mitotic state. When the stent is deployed, IEL agents are removed near the stent struts due to high stress. Consequently, neighboring SMCs enters the synthetic phase<br>- NO<threshold induces proliferation; NO>threshold stops proliferation<br>- NO concentration is computed based on WSS and on the presence of functional endothelium. Assumption of complete endothelium denudation after PTA and presence of functional endothelium over time after stenting from literature<br>- Scenarios of re-endothelialization either from the boundaries of the stented region or random<br>- Strained synthetic SMCs produce ECM at a constant rate until they switch back to contractile phenotype<br>- SMCs produced ECM stochastically (based on 50-80% of ECM in the neointima as reported in literature): Probability=0.1 per hour per SMC<br>- ECM agents follow the same mechanical interaction rules as SMC<br>- ECM placed away from the lumen to mimic the chemotactic migration of SMC towards the lumen<br>- IEL and EEL with different mechanical formulation to simulate laminae retraction when break
Boyle et al. (2010)	In-stent restenosis	Endothelium, arterial wall (single compartment)	SMC, EC<br>ECM, MDF, GF modeled as agents' internal variables	- Initialization: ECM=1, random distribution of SMC<br>- Lattice with minimum principal stress > threshold: SMC are removed and GF and MDF introduced<br>- EC cover the lumen surface except for the stented region (total denudation)<br>- Contractile SMCs switch to synthetic phenotype if the ECM concentration is below a threshold and SMC/ECM ratio is not appropriate<br>- SMC proliferation at constant rate if GF>threshold. SMC proliferation reduces GF<br>- SMC random migration at constant rate, in lattice points with ECM or close to ECM.<br>- SMC cannot occupy a position containing another cell (contact inhibition)<br>- SMC produce ECM at constant rate in their lattice position<br>- ECM degradation in lattice points with ECM and MDF: both components are reduced as ECM is degraded (constant rate)<br>- EC occupy lattice without ECM and close to a position with ECM<br>- EC proliferate on the lumen surface at constant rate. Once it heals it constitutes a physical barrier to SMC proliferation
Boyle et al. (2011)	In-stent restenosis	Arterial wall (single compartment)	SMC<br>ECM, MDF, GF and DA, modeled as agents' internal variables	- Set of ODEs quantifying ECM, MDF, GF and DA at each time step<br>- SMC phenotype as continuum variable in the range 0-1, modulated by ECM<br>- Contact-inhibition: SMC proliferates or migrates only if there's a vacant neighboring lattice site<br>- Probability SMC mitosis as function of: maximum proliferation rate, GF, phenotype<br>- SMC migration: random towards empty sites with ECM>threshold. Probability of SMC migration depends on maximum velocity and phenotype<br>- SMC production of e described by ODE

Zahedmanesh et al. (2014)	In-stent restenosis	Endothelium, arterial wall (single compartment)	SMC, EC<br>ECM, MDF and DA modeled as agents' internal variables	Initialization:<br>- Random seeding of quiescent SMC, according to physiological density<br>- ECs removed in the damaged area (near the strut)<br>- Physiological density of collagen<br>Rules:<br>- DA upregulates MMP synthesis by SMC<br>- MDF degrades ECM. As ECM is degraded also DA decreases<br>- SMC switch to synthetic phenotype if ECM<threshold (value in healthy arteries). If the DA stimulus recessed and ECM>threshold they switch back to contractile phenotype<br>- SMC proliferates and migrates in empty sites (CI)<br>- SMC produce ECM at constant rate<br>- A maximum SMC density is allowed, beyond which proliferation stops<br>- Presence of ECs within a radius of 60 µm lead to SMC switch back to contractile phenotype, even if ECM<threshold (to simulate the quiescent effect of NO)<br>- ECs can only proliferate on the lumen surface until complete re-endothelialization
Nolan and Lally (2018)	In-stent restenosis	Endothelium, arterial wall (single compartment)	SMC, EC<br>ECM, MDF, GF phenotype and DA modeled as agents' internal variables	Initialization:<br>- ECM, MDF, DA and GF quantified at each time step for each agent (comparing two damage models)<br>- Random seeding of quiescent SMC, according to physiological density<br>- ECs removed in the damaged area (near the strut)<br>- Physiological density of collagen<br>Rules:<br>- SMC proliferate if 3 criteria hold: it is a synthetic phenotype, the doubling time probability is > random generated number, there is an empty neighboring space (contact-inhibition)<br>- Presence of ECs within a radius of 60 µm lead to SMC switch back to contractile phenotype, even if ECM<threshold (to simulate the quiescent effect of NO)<br>- ECs proliferate with a fixed doubling time of 92h
Li et al. (2019)	In-stent restenosis	Endothelium, arterial wall (single compartment)	SMC, EC<br>ECM, MDF, GF phenotype and DA modeled as agents' internal variables	Initialization:<br>- ECM, MDF, GF and DA computed through a set of ODEs<br>- ECs removed from the lumen surface<br>Rules:<br>- SMC proliferation as function of ECM, MDF and DA as in (Boyle et al. 2011)<br>- SMC switch to contractile if EC is closer than 60 µm<br>- Contact inhibition: agents are not allowed to overlap<br>- ECs are allowed to proliferate if they have only one neighboring EC
Garbey et al. (2015)	Vein graft remodeling	Intima, media layers	SMC, ECM	- SMC proliferation in the intima as function of WSS<br>- Constant SMC apoptosis in the intima<br>- Constant SMC proliferation in the media<br>- SMC apoptosis in the media as function of the wall tension<br>- SMCs migration from the media to the intima as function of WSS and wall tension<br>- Constant ECM production in the intima<br>- ECM degradation in the intima as function of WSS<br>- Constant ECM production in the media<br>- ECM degradation in the media as function of WSS

Garbey et al. (2017)	Vein graft remodeling	Intima, media layers	SMC, ECM	- SMC proliferation in the intima as function of WSS<br>- SMC proliferation in the media as function of wall tension<br>- Constant SMC apoptosis in the intima and media layers<br>- ECM degradation in the intima as function of WSS<br>- ECM degradation in the media as function of wall tension<br>- Constant ECM production in the intima and media layers
Garbey et al. (2019)	Vein graft remodeling	Intima, media layers	SMC, ECM	- SMC proliferation in the intima as function of WSS<br>- Constant SMC apoptosis in the intima<br>- ECM production in the media as function of wall tension<br>- Constant ECM degradation in the media<br>- SMCs migration from the media to the intima as function of WSS
Zahedmanesh et al. (2012)	Remodeling of a vascular tissue-engineered scaffold	Single layer	SMC, ECM	Initialization:<br>- One row of SMCs to the luminal and abluminal surfaces<br>Rules:<br>- SMC random migration<br>- SMC mitosis: it occurs if the cell reaches its doubling time and the contact inhibition criterion holds (vacant surrounding site). Doubling time is function of cyclic strain and pore fluid velocity: doubling time increases with cyclic strain and decreases with pore fluid velocity<br>- SMC apoptosis: probability function depending on cyclic strain and pore fluid velocity. Probability linearly increases with cyclic strain and decreases with pore fluid velocity (=0 at a certain threshold of pore fluid velocity). A random number is generated for each cell and compared with the probability: when the logical statement is true, apoptosis occurs<br>- ECM production: at each time step each SMC deposited ECM. The amount of ECM deposited is linearly dependent on cyclic strain
Keshavarzian et al. (2018)	Arterial growth and remodeling under different conditions: growth factors, chemicals, blood pressure	Intima media and adventitia layers	EC, SMC, fibroblasts, ECM	Each lattice site can be occupied by a number of cells and a proper amount of ECM and soluble factors.<br>Rules:<br>- SMC proliferation as function of platelet-derived growth factor<br>- SMC apoptosis (constant)<br>- SMC production of platelet-derived growth factor (stretch induced), matrix metalloproteinases (constant/stretch induced) and collagen (tissue growth factor-β dependent)<br>- EC production of NO, platelet-derived growth factor and endothelin-1 (flow dependent)<br>- matrix metalloproteinase reduction of ECM (collagen, elastin, gelatin), based on matrix metalloproteinases content<br>- matrix metalloproteinase removal (function of matrix metalloproteinase content)<br>- Fibroblast proliferation (function of platelet-derived growth factor)<br>- Fibroblast apoptosis (constant)<br>- Fibroblast production of collagen (stretch induced)<br>- Fibroblast production of matrix metalloproteinases (constant)

	-	If a lattice site surpasses its nominal volume, then a random cell (SMC, EC or AF) is chosen to migrate to a neighboring site whose volume is lower than the nominal one. This is performed until the site restores a proper volume

ODE: ordinary differential equation; PDE: partial differential equation; IBM: immersed boundary method; I: input; O: output; WSS: wall shear stress; OSI: oscillatory shear index; SMC: smooth muscle cell; EC: endothelial cell; ECM: extracellular matrix; LDL: low density lipoprotein; IEL: internal elastic lamina; EEL: external elastic lamina; NO: nitric oxide; CI: contact inhibition; MDF: matrix degrading factor; GF: growth factor; DA: damage; 2D: bidimensional; 3D three-dimensional.
